# Predicting gene expression state and prioritizing putative enhancers using 5hmC signal

**DOI:** 10.1186/s13059-024-03273-z

**Published:** 2024-06-03

**Authors:** Edahi Gonzalez-Avalos, Atsushi Onodera, Daniela Samaniego-Castruita, Anjana Rao, Ferhat Ay

**Affiliations:** 1 La Jolla Institute for Immunology, 9420 Athena Circle, La Jolla, CA 92037 USA; 2 Bioinformatics and Systems Biology Graduate Program, University of California San Diego, La Jolla, CA 92093 USA; 3 Department of Immunology, Graduate School of Medicine, Chiba University, Chiba, 260-8670 Japan; 4 Biological Sciences Graduate Program, University of California San Diego, La Jolla, CA 92093 USA; 5 Department of Pharmacology, University of California San Diego, La Jolla, CA 92093 USA; 6 Sanford Consortium for Regenerative Medicine, La Jolla, CA 92093 USA; 7 Moores Cancer Center, University of California San Diego, La Jolla, CA 92093 USA; 8 Department of Pediatrics, University of California San Diego, La Jolla, CA 92093 USA

## Abstract

**Background:**

Like its parent base 5-methylcytosine (5mC), 5-hydroxymethylcytosine (5hmC) is a direct epigenetic modification of cytosines in the context of CpG dinucleotides. 5hmC is the most abundant oxidized form of 5mC, generated through the action of TET dioxygenases at gene bodies of actively-transcribed genes and at active or lineage-specific enhancers. Although such enrichments are reported for 5hmC, to date, predictive models of gene expression state or putative regulatory regions for genes using 5hmC have not been developed.

**Results:**

Here, by using only 5hmC enrichment in genic regions and their vicinity, we develop neural network models that predict gene expression state across 49 cell types. We show that our deep neural network models distinguish high vs low expression state utilizing only 5hmC levels and these predictive models generalize to unseen cell types. Further, in order to leverage 5hmC signal in distal enhancers for expression prediction, we employ an Activity-by-Contact model and also develop a graph convolutional neural network model with both utilizing Hi-C data and 5hmC enrichment to prioritize enhancer-promoter links. These approaches identify known and novel putative enhancers for key genes in multiple immune cell subsets.

**Conclusions:**

Our work highlights the importance of 5hmC in gene regulation through proximal and distal mechanisms and provides a framework to link it to genome function. With the recent advances in 6-letter DNA sequencing by short and long-read techniques, profiling of 5mC and 5hmC may be done routinely in the near future, hence, providing a broad range of applications for the methods developed here.

**Supplementary Information:**

The online version contains supplementary material available at 10.1186/s13059-024-03273-z.

## Background

5-methylcytosine (5mC) is a covalent DNA modification and DNA epigenetic mark that is deposited *de novo* by DNA Methyltransferases 3A (DNMT3A) and 3B (DNMT3B) and maintained during DNA replication by the DNMT1/UHRF1 maintenance methyltransferase complex [1, 2]. The mammalian Ten-Eleven Translocation (TET) family of dioxygenases is comprised of TET1, TET2, and TET3, which oxidize 5mC to 5-hydroxymethylcytosine (5hmC), 5-formylcytosine (5fC), and 5-carboxylcytosine (5caC) [3–8]. These three oxidized methylcytosines are essential intermediates in all known mechanisms of DNA demethylation [9–11].

We and others have developed immunoprecipitation and capture assays, including GLIB-seq [12], CMS-IP [13], hMe-Seal [14], nano-hmC-Seal Han [15], optical 5hmC mapping [16], hMEDIP [17] and HMCP [18, 19], to survey 5hmC signal genome-wide. Independent of the method used, 5hmC is consistently associated with active genomic regions or “epigenetically dynamic loci” [20, 21]. 5hmC is particularly enriched in active cell-specific enhancers [20, 21] which bind transcription factors (TFs) that regulate expression of the genes controlled by those enhancers. Enhancers that are newly activated during cellular activation or differentiation show progressive deposition of 5hmC and loss of 5mC during activation and differentiation [19]. 5hmC is a highly stable modification in differentiated non-proliferating cells [22]. 5hmC is also strongly enriched in accessible genomic regions [19, 23], as well as in euchromatin and transcribed regions [24, 25].

In addition to its enrichment at active enhancers, 5hmC is enriched in the gene bodies (or genic region and vicinity) of highly expressed genes. T cells and their precursors have high 5hmC levels across the gene body and Transcription Termination Sites (TTS) but lower 5hmC levels at their transcriptional start sites (TSS), because these generally also have low levels of the parental base, 5mC [9, 20, 21]. This pattern of 5hmC enrichment has also been observed in multiple other cell types, including embryonic stem cells [20], neurons [26], cardiomyocytes [27], colon epithelia [28], liver [29], myeloid and megakaryocytic erythroid progenitors [30], and others [15, 31].

The pattern of 5hmC enrichment at actively transcribed gene bodies and active enhancers suggested that we might be able to use 5hmC alone to predict gene expression patterns across the genome. An extensive number of previous approaches have attempted to predict gene expression values or state (high/low, on/off) from DNA sequence alone [32–34], from methylation information [35], from markers of chromatin accessibility [36], from landmark genes [37], and by integration of multiple histone marks [38, 39]. These methods have made use of powerful machine learning techniques, including more recent deep learning architectures [40–42]. For example, DeepChrome [38], used five histone H3 marks (H3K4me1, H3K4me3, H3K9me3, H3K27me3, and H3K36me3) to train a deep neural network in a binary classification task to predict high versus low expression of genes in 56 different cell-types using the REMC database [43], with an average AUROC/AUC (area under the receiver operating characteristic curve) of 0.8. More recently, Enformer [44] was developed to predict gene expression from DNA sequences by integrating information from flanking regions in the genome up to 100 kb away from the gene of interest and achieved a correlation of 0.85 in predicting CAGE (cap analysis gene expression) signal at the TSS of human protein-coding genes.

Many of the above-mentioned methods for gene expression prediction use a vast amount of data. Here, we first developed a deep convolutional network model (DNN) that by utilizing only 5hmC enrichment in genic regions and their vicinity was able to predict gene expression state (high/low) with an AUC of 0.87 across 49 different cell types. This predictive performance was robust to different train/test splits in a leave-one-out setting across the 19 autosomal chromosomes of the mouse genome. In addition, the developed DNN model generalized to unseen chromosomes of the unseen cell types that were held out from the training (average AUC of 0.86). By decomposing the output prediction using DeepLift [45], we observed that both positive and negative contributions to expression prediction tasks were highest for the 500-bp region that is immediately downstream of the TSS region and inside the gene body.

In addition, numerous studies have used epigenetic marks as tools to link regulatory regions such as enhancers to their target gene(s). Most of these studies have focused on signals such as histone marks (H3K27ac, H3K9me3, etc.), accessible genomic regions based on assay for transposase-accessible chromatin sequencing (ATAC-seq) [46, 47], or more recently, chromosome conformation capture methods such as Hi-C or its variants [48]. The Activity by Contact (ABC) model [48] scores enhancer-gene connections to predict enhancers and their target genes by the use of Hi-C contact frequencies (chromatin conformation) and chromatin accessibility or histone acetylation. TargetFinder [49] models the interaction status of predefined pairs of enhancers and promoters by integration of multiple genomic features. Other notable attempts at modeling gene regulation and predicting gene expression utilizing 3D genome organization include GC-MERGE [50], GraphReg [51, 52], and E2G [53]. A key component of some of these models is the use of more complex machine learning operations such as graph-structured data to develop “graph convolutional networks” (GCNs; [54]), which can produce representations that encode both local graph structure (connectivity) and features of nodes, known as vector embeddings (or simply “embeddings”). Instead of training individual embeddings for each node, GraphSAGE, a novel approach introduced by Hamilton and colleagues [55], learns an aggregation function that synthesizes feature information from a node's immediate network vicinity to efficiently produce vector embeddings. Once trained, this function is adept at generating embeddings for previously unseen data, thus extending its utility to datasets beyond the scope of its initial training.

Considering the observed 5hmC enrichment in cell-specific distal enhancers, we were interested in integrating 5hmC with 3D chromatin structure data to prioritize putatively functional enhancer regions for each gene while performing the task of predicting that gene’s expression state. For this, we started with adapting the recently developed Activity-by-Contact (ABC) model [48] to utilize the 5hmC signal (ABC-5hmC) instead of H3K27ac (ABC-H3K27ac). For activated B cells, ABC-5hmC captured >89% of the regions identified as putative enhancers by ABC-H3K27ac but also reported over 17,000 additional regions with strong 5hmC signal and weaker ATAC-seq peaks. One of the putative elements uniquely captured by ABC-5hmC corresponded to a region that shared 5hmC dynamics with two other validated TET-dependent enhancers of the *Aicda* gene, the primary regulator of class switch recombination (CSR). On the other hand, ABC-H3K27ac-specific regions were enriched for H3K4me3 signals and TSS proximity.

As another way of integrating one-dimensional 5hmC signal enrichment with chromatin contact maps, we trained graphical 5hmC convolutional networks (“GhmCNs”) to also predict gene expression state (high/low). To achieve this, we used the graphical convolutional network structure developed by Bigness and colleagues [50]. This structure makes use of the GraphSAGE framework [55], which allowed us to train an embedding-generator function on one cell-type, and then to use this function in a previously unseen cell type. We demonstrated the power of our approach (GhmCN model) using graph structures generated from cell-type-specific and aggregate contact maps (all in 10 kb resolution) to predict gene expression state across six different cell types. By decoding the trained models with GNNExplainer, we prioritized putative regulatory regions containing 5hmC-rich stretches, some of which have been previously validated in the literature as functional enhancers. For genes of specific importance to the immune cell types examined, we reported regions that bore several hallmarks of *bona fide* enhancers such as chromatin accessibility, transcription factor binding sites (TFBS), and physical binding of TFs as measured by ChIP-seq. Our studies provide novel methods for predicting gene expression status and putative regulatory elements together with their target genes primarily from 5hmC, an intrinsic epigenetic modification of DNA that can be measured and mapped without a requirement for intact viable cells.

## Results

### 5hmC features across gene body are predictive of gene expression state

**Fig. 1:**
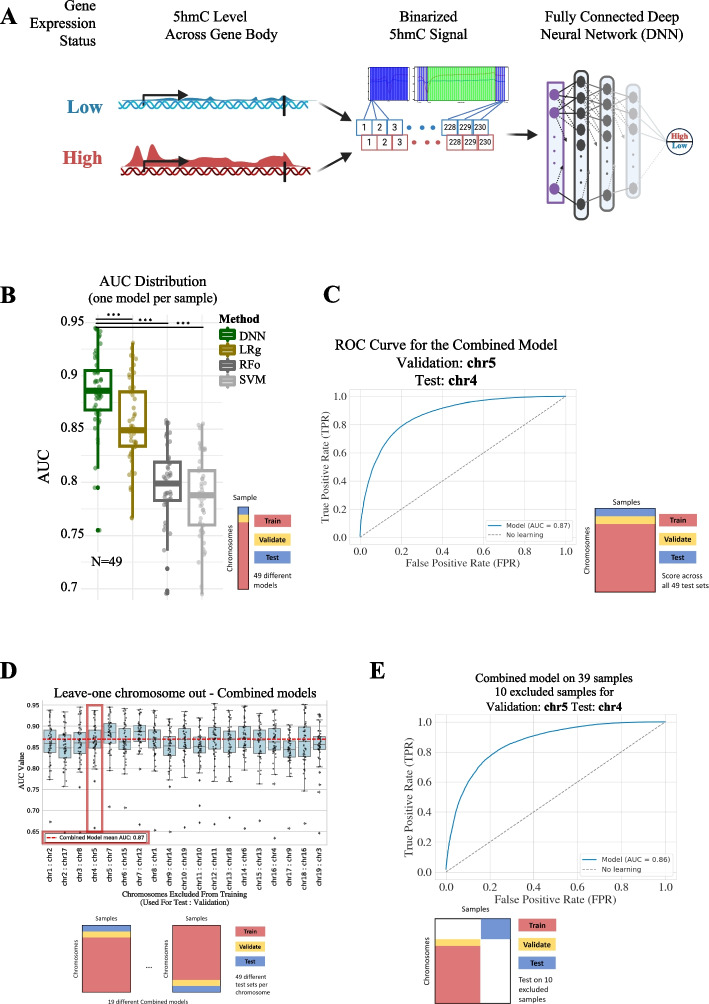
Evaluation of different methods to predict gene expression state from 5hmC signal. **A** Schematic of our 5hmC-based (normalized signal) feature extraction across the gene body, upstream of the transcription start site (TSS), and downstream of the transcription termination site (TTS) to train machine learning models including the fully connected deep neural network (FCDNN) we develop in this work. **B** Area under the receiver operating characteristic curve (AUC) distribution for our FCDNN model and baseline machine learning models: logistic regression (LRg), random forest (FRo), and SVM. For this analysis, we train one model per sample while holding out one chromosome for validation/development and one chromosome for testing. Statistical significance testing across different models was performed using the Wilcoxon rank sum test with *** indicating a *p*-value less than 1e − 8. **C** ROC curve of a combined FCDNN model trained using all 49 datasets (“combined model”) with a schematic of the data split used for training, validation, and testing. **D** AUC score distributions to assess the robustness of the combined model approach by leaving out a different chromosome for testing each time. We trained 19 different models each with a different set of excluded test and validation chromosomes, indicated in the *X*-axis. Each box plot shows the distribution of the AUC scores calculated for the test chromosome across 49 different samples. The combined model with the ROC curve reported in panel **C** is highlighted with a red box and its overall AUC is depicted by the horizontal dashed line. **E** ROC curve of the combined model to assess whether the trained models generalize to unseen cell types. We trained a combined model on a subset of chromosomes for the 39 samples and tested on an unseen test chromosome of 10 samples that are excluded from training as depicted by the schematic

We compiled paired sets of 5hmC-immunoprecipitation sequencing (CMS-IP-seq, hMEDIP, HMCP, GLIB-seq, hMe-Seal, and their matched input samples) and RNA-seq data for 153 replicate experiments (Additional file 2: Table S1–S4). After quality control and selection of one representative replicate for each experimental condition, we kept 49 samples to develop our predictive models (Methods). For each sample, we obtained 5hmC signal per bin using 5hmC enrichment versus input (normalized for sequencing depth and bin size). For each gene over 1 kb in size (n=21,752), we selected a total of 230 5hmC features using fixed and variable-sized bins across the gene body and around the TSS and TTS (Methods, Additional file 1: Fig. S1A–B). For the same set of genes, we categorized their expression state into two groups (high vs low) using the median value of gene expression for that sample (Methods, Additional file 1: Fig. S1C). Our analysis of variance of expression across genes ranked by TPM values for each sample indicated that our dichotomization roughly separates genes into two regimes with high variation genes labeled as Low expression and genes with low expression variation labeled as High (Additional file 1: Fig. S1D). We then developed predictive models using these 5hmC features and expression labels with different training/validation/test splits across samples and across chromosomes (Fig. 1A). In each setting, in order to avoid effective memorization of average values by our models, a pitfall highlighted in gene expression prediction tasks [56], we withheld whole chromosome(s) from the training to evaluate our predictions in a truly unseen set of genes.

We first assessed whether 5hmC can be utilized by traditional machine learning approaches and a deep neural network model to predict gene expression state when trained and tested with data from a single sample. For each of our 49 samples, we trained three models (logistic regression (LRg), support vector machines (SVM), and random forest models (RFo)) using well-established machine learning methods that can be used off-the-shelf through commonly used software packages [57, 58]. In addition, we developed a fully connected deep neural network (FCDNN or DNN) as such models provide powerful approximations to complex functions linking input features to output labels [59]. For this analysis, we trained each model using all chromosomes except chr5 for validation and chr4 for testing. To evaluate the performance of the trained models, we calculated the area under the curve (AUC) scores from the receiver operating characteristic (ROC) for the test set. Under default parameters (see the “ Methods” section), we found that 5hmC signals displayed predictive power with the three conventional machine learning methods (median AUC values 0.85, 0.8, and 0.79 for LRg, RFo, and SVM, respectively, Fig. 1B and Table 1) and that the predictive power varied across different cell type (0.7 to 0.93 — Additional file 2: Table S5). We then trained FCDNNs for the same predictive task using the same 5hmC input features and the same train/validate/test split. Using the validation set, we first selected hyperparameters such as the number of layers and neurons per layer (Table 2). We then compared the resulting FCDNN models and observed that they significantly outperform the three machine learning approaches discussed above (Fig. 1B) with a median AUC of 0.89 across all samples (row “Sample-specific” AUCs in Table 3 and F1 scores in Table 4 with per sample statistics in Additional file 2: Table S5 and Additional file 2: Table S6, respectively).

**Table 1:**
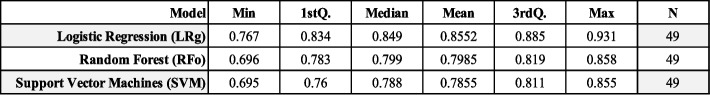
AUC score distribution for each traditional machine learning tool on the gene expression prediction task

**Table 2:**
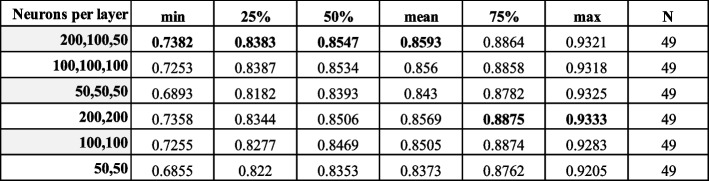
Hyperparameter tuning of total connected layers and neurons per layer. Shown are the summary statistics values across all AUC scores per sample per configuration (using validation dataset)

**Table 3:**
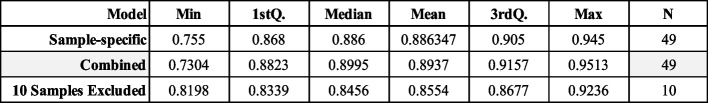
Summary statistics of the AUC scores per DNN model processing each sample’s unseen test datasets “Final results”

**Table 4:**
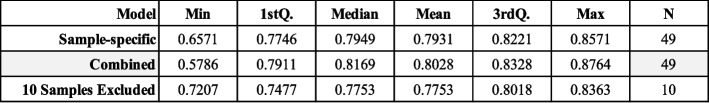
Summary statistics of the F1 scores per DNN model processing each sample’s unseen test data

### Predictive models of gene expression from 5hmC are generalizable across cell types

Next, we developed a combined model that utilized training data from all 49 samples to predict expression state for genes from an unseen chromosome. Similar to within sample models, we first started with holding out chr5 for validation and chr4 for testing such that the model does not see these chromosomes for any of the samples. When evaluated using chr4 genes concatenated across all samples, we obtained an AUC of 0.87 for this combined model (Fig. 1C). We then asked to whether this performance was robust to choices of test/validate/train split and, to assess that, we developed 19 different combined models with each one setting aside a different chromosome for testing and a random (sampled without replacement) chromosome for validation. Our results showed that predictive performance was quite robust across these different models (Fig. 1D) suggesting minimal impact with respect to which chromosome(s) are held out from the training (row “Combined” AUCs in Table 3 and F1 scores in Table 4 with per sample statistics in Additional file 2: Table S5 and Additional file 2: Table S6, respectively).

For the above experiments, the training and test sets were still contributed by each cell type. In order to better assess the generalizability of our predictions to completely unseen cell types, we repeated our training by withholding a number of samples from the training set (*n* = 10) and using them as test sets in the final AUC calculation. Due to the robustness of the combined models which we discussed above, we chose to use only one model by holding out chr5 for validation and chr4 for testing as before. From this, we obtained an overall AUC of 0.86 for the set of test genes concatenated across all 10 excluded samples (Fig 1E; row “10 Samples Excluded” AUCs in Table 3 and F1 scores in Table 4 with per sample statistics in Additional file 2: Table S5 and Additional file 2: Table S6, respectively). These results suggest that our predictive models generalize well to cell types or samples that have not yet been seen by the model. Such generalization may allow us to have an approximate gene expression profile for non-viable samples with no available RNA or protein but sufficient DNA to profile 5hmC enrichment.

### Further assessment of our predictive models and potential confounding factors

To better characterize the predictive performance of our models, for each sample, we divided genes into four quartiles with respect to their expression (TPM) such that Q4 has the top 25% of genes with the highest expression. We then assessed our model in correctly predicting High/Low expression for each quartile. Although the median accuracy was over 0.9 across all samples for genes with lowest (Q1) and highest (Q4) expression it dropped to 0.71 and 0.73 for middle quartiles, highlighting the difficulty of binarizing the expression state of genes with intermediate levels of expression (Additional file 1: Fig. S2A).

Another assessment we conducted was to consider the variability of gene expression and expression states across different samples and how it impacts prediction accuracy. For this, we used a simple baseline that “memorizes” expression state across training samples to predict a label for a held-out sample using a simple majority vote (e.g., 30 high, 18 low labels across 48 training samples leads to a prediction of High for that gene for any unseen sample). By definition, this model will be 100% accurate for genes that are always High (e.g., housekeeping genes) or Low across all samples. Therefore, we focused on genes with variable labels across our samples to compare our combined DNN models to this majority vote baseline. For genes whose expression state shows any variation, our model outperforms the baseline with a median accuracy of over 78% versus 68% across all samples (Additional file 1: Fig. S2B). We observed a similar but more striking difference for the genes whose expression state is the most variable across samples (genes whose underrepresented label covering at least a third of the samples (Additional file 1: Fig. S2C)). These results suggest that our models effectively utilize cell-type-specific 5hmC patterns to predict gene expression labels for genes that have cell-type-specific activity.

One other important factor that may impact our predictions is the sequence decomposition differences across genes with different expression patterns and, especially, across promoter regions of such genes. To evaluate this, we categorized genes into five non-mutually exclusive groups with respect to their gene expression values (e.g., TPM = 0 across all samples), states (e.g., most variable, always high or always low), and previous annotations (e.g., Ubiquitously expressed across mouse tissues). We then compared the CpG content distributions of promoter regions (+/− 1 kb around the TSS) for these groups and observed substantial differences (Additional file 1: Fig. S2D). As previously documented [60], we observed that the CpG content of the promoter has a positive correlation with gene expression (e.g., highest overall CpG content for genes labeled Always High). However, since we avoid memorization of constitutive features in our DNN model by leaving out entire chromosomes from the training, this sequence content bias does not become an obvious pitfall for our approach. Our above-mentioned performance for genes with variable expression states across samples also suggests our model’s ability to incorporate the cell-type-specific modification information as intended. Given the above findings concerning the importance and contribution of cell-specific and sequence-based features of the promoter regions, we performed one last evaluation by removing any bin surrounding the promoter region (130 total bins surrounding TSS) from the 5hmC feature set. Although we observed a drop in predictive performance when bins surrounding the TSS are hidden from the model training (accuracy from 0.79 to 0.73 and AUC from 0.87 to 0.83), there remains substantial predictive power in 5hmC features of bins representing the gene body independent of the promoter region.

### Decoding the deep learning models identifies 5hmC features most predictive of gene expression

**Fig. 2:**
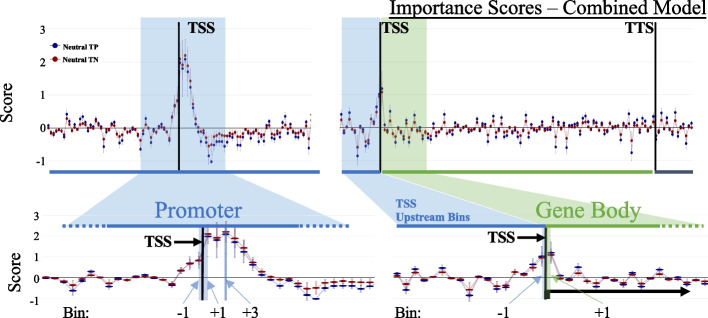
Decoding deep neural network predictions. (Top) Distribution of DeepLift significance scores of the combined model throughout the 230 bins, using a neutral combination of input signal for network activation and decoding. (Bottom) A zoomed-in version of the 5hmC feature bins and their contribution scores across promoter/TSS bins (Left) and all bins (right). Blue indicates fixed-sized bins (100 bp) and green indicates the variable-sized gene body bins

To define the most important 5hmC features/patterns in performing the gene expression prediction task, we implemented DeepLift [45], a tool that gives a contribution score to each of the features of a DNN, relative to the state of the network after a “reference” signal (e.g., any gene’s 5hmC signal distribution) is processed by the network. To obtain a distribution of relative contribution per feature, we fed DeepLift the networks activated by neutral signal (Methods). This neutral signal was generated using randomly sampled genes (an equal number of high and low genes) and averaging their signal for each of the 230 bins. We decoded the combined model for both labels (“high” and “low”) and found that the features representing the TSS, and those surrounding the promoter, have the highest feature importance (Fig. 2). For fixed-size bin representation of the promoter region, the first 500 bps downstream of TSS had the highest contribution scores, whereas for the variable sized bins representing gene body it was the very first bin downstream of TSS that represents 1% of the gene’s span. These results are consistent with previous studies finding that the signals slightly downstream of TSSs are the most informative [61], and that epigenetic features in or near the promoter region were the most informative in the gene expression prediction task [38, 39, 62]. These results may reflect contributions from downstream promoter elements (DPE) that are conserved from Drosophila to humans and bind transcriptional activators such as TFIID [63, 64].

### 5hmC-based Activity-by-Contact model identifies novel distal enhancers and their target genes

**Fig. 3:**
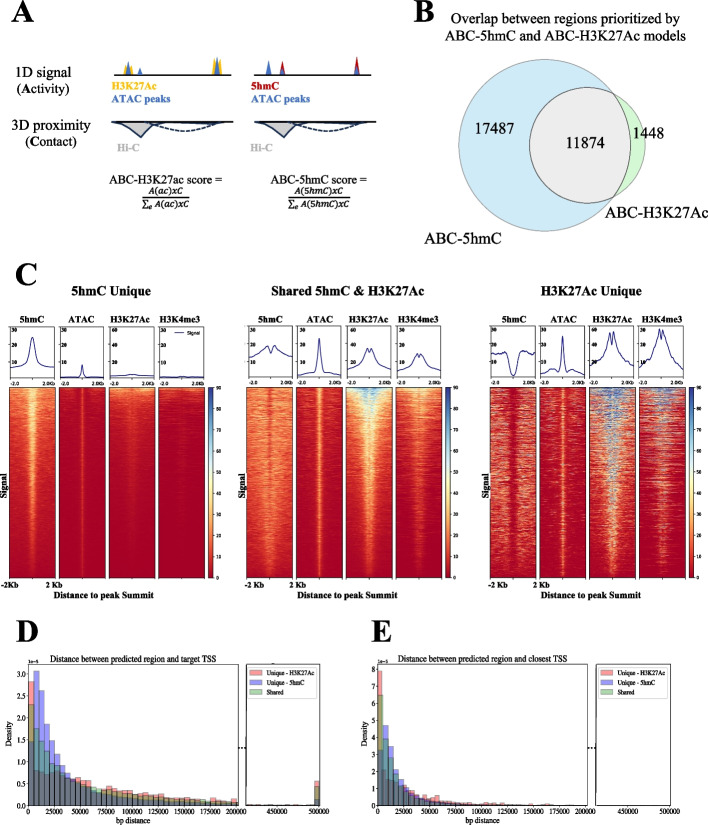
Activity-by-contact (ABC) model using 5hmC versus H3K27ac. **A** Schematic representation of the published ABC (referred to here as ABC-H3K27ac) and our new ABC-5hmC model. Both models use ATAC-seq peak regions as “candidate enhancers” and the same Hi-C data for computing the contact score. **B** Venn diagram between ABC-5hmC and ABC-H3K27ac prioritized regions using data from activated B cells (72 h). ABC-5hmC captured most of the regions prioritized as putative enhancers by ABC-H3K27ac. **C** Tornado plots for the three different sets of regions from the Venn diagram in panel **B**. A bin size of 10-bp was used and + / − 2-kb region around the ATAC-seq peak summits was plotted for 5hmC, ATAC-seq, H3K27ac, and H3K4me3 signals for the activated B cells. **D**–**E** The density histograms of genomic distances between ABC-prioritized regions and their target gene TSSs (**D**) or to the closest gene TSS (**E**) for the three different sets of regions in panels **B** and **C**

Given the robust gene expression predictions drawn from using only 5hmC signal enrichment as a 1D epigenetic mark using low-complexity neural network structures, and considering the observed 5hmC enrichment in cell-specific distal enhancers [20, 21], we hypothesized that integration of 5hmC signals with 3D chromatin organization would allow us to predict putatively functional enhancer regions for each gene. To test this, we employed a popular recent approach that combines enhancer activity (usually measured by H3K27ac) with the amount of contact between a putative regulatory region and its potential target gene (usually measured by Hi-C), namely the Activity-by-Contact (ABC) model [48]. We adapted ABC model such that it utilizes 5hmC signal (ABC-5hmC) and compared the resulting predictions of enhancer-promoter links to those from the original ABC model that uses H3K27ac (ABC-H3K27ac) (Fig. 3A). We performed this comparison for activated mouse B cells for which we had gene expression from RNA-seq, H3K27ac enrichment from ChIP-seq, 5hmC enrichment from CMSIP and chromatin accessibility from ATAC-seq from our earlier work [19]. We also gathered and processed the high-depth Hi-C data from [65] and processed it at 10 kb resolution (Methods).

Using ATAC-seq peaks as the starting point for both ABC models, we showed that ABC-5hmC identified over 29,000 putative enhancer regions linked to 10,442 different genes. Among these were nearly 12,000 regions that were shared with ABC-H3K27ac predictions, which constituted over 89% of all regions reported by ABC-H3K27ac, linked to 8788 different genes (Fig. 3B). We further assessed the common and unique sets of regions across the two models using aggregate plots and heatmaps for 5hmC, ATAC-seq, H3K27ac and H3K4me3 enrichment at and nearby these regions (Fig. 3C). The 11,874 shared regions (Fig. 3C, *center panel*) all showed a strong signal for ATAC-seq (as expected) and strong aggregate signals for both H3K27ac and H3K4me3 in the immediate vicinity of these ATAC peaks (the local dip in the middle for histone modifications is due to nucleosome-free regions). Further inspection of the histone modification enrichments suggests that a subset of regions (top portion) have prominent H3K4me3 signal and this same set also has a local depletion of 5hmC signal due to the paucity of the TET substrate 5mC, all suggestive of overlap with, or proximity to, active CpG-rich gene promoters. The 1,448 regions unique to ABC-H3K27ac model showed similar patterns (e.g., H3K4me3 enrichment) with much more pronounced depletion of 5hmC at their center across almost all regions, suggesting that this set is mainly composed of active promoters (Fig. 3C, *right panel*). It is well known that promoters with active chromatin states serve as enhancers to other distal genes [66, 67]; hence, ABC-H3K27ac-unique regions are likely participating in such promoter-promoter interactions. In contrast, ABC-5hmC unique regions (by definition with high enrichment of 5hmC) did not have any enrichment for H3K4me3 or of H3K27ac (Fig. 3C, *left panel*). The ATAC-seq enrichment for ABC-5hmC regions was weaker compared to regions common to both ABC models or specific to ABC-H3K27ac.

These findings suggest ABC-5hmC model might be picking up distal interactions with weak enhancers or with latent enhancers that are unmarked and unbound in the absence of a specific stimulus [68]. Primed enhancers defined by the presence of H3K4me1 and lack of H3K27ac [69–71] could have been another possibility, however, we observed no H3K4me1 enrichment with published data albeit QC metrics and enrichment scores demarcated these ChIP-seq samples as low quality [72]. This set of regions with strong 5hmC may also correspond to a new class of regulatory elements that work in conjunction with classical enhancers (one example would be the recently proposed facilitator elements [73]). The distance distribution between predicted regions and their target gene’s TSS show that while ABC-H3K27ac specific predictions are enriched for very short- (within 5 kb) and very long-range interactions (> 500 kb), ABC-5hmC predictions show a preference for mid-range interactions (greater than 5 kb but less than 40 kb) (Fig. 3D). When we plotted a similar distance distribution for the closest gene TSS rather than the TSS of the ABC-predicted target gene, we also see a strong enrichment for predictions being within 5 kb of a TSS for ABC-H3K27ac compared to ABC-5hmC (Fig. 3E), which supports our observations that the ABC-H3K27ac model preferentially identifies interactions with other promoters.

### Integrating distal 5hmC signals in the prediction of gene expression using graph convolutional network (GCN)

**Fig. 4:**
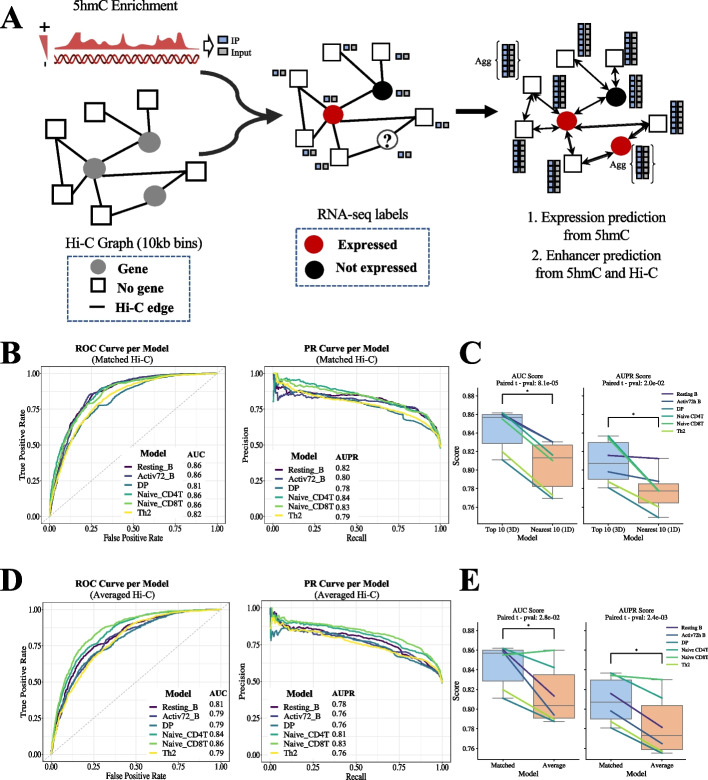
A graph convolutional network approach to utilize 5hmC for predicting expression state and for prioritizing putative regulatory regions. **A** Schematic of our GhmCN model. By splitting the mouse genome in 10-kb windows and using Hi-C data, we generated the network structure with each node connected to their top-10 neighbors with respect to normalized Hi-C contact strength. Each node (10-kb window) is associated with a single measurement of 5hmC immunoprecipitation (IP) and its respective control (input signal) depicted by small squares. The aggregate function “agg” is implemented to all nodes during convolutions in training but illustrated only in a couple of nodes in the schematic for clarity. The graph convolution network was then trained based on the labels of nodes where the TSS of a gene was present. **B** Evaluation metrics (ROC and PR curves) for each of the six models trained and tested using a matching set of Hi-C, 5hmC signal, and expression information per cell type. DP and Th2 cell types had the lowest scores, likely due to the low sequencing depth of their Hi-C contact maps. **C** AUC and AUPR scores to assess whether Hi-C data contributes significantly to the model performance as opposed to simply using 10 nearest bins to the TSS for each gene (i.e., 5 upstream and 5 downstream bins of TSS). **D** Evaluation metrics for each of the six models were trained and tested using an averaged set of Hi-C contacts (Hi-C data from each cell type was subsampled to the same number of valid interaction pairs before aggregation) but with cell-specific 5hmC signal. All samples performed better when using cell-type-specific data with the performance gap being higher for cell types with the highest depth Hi-C data (i.e., B cells with 1B + valid interactions). **E** AUC and AUPR scores to assess whether cell-specific Hi-C data contributes significantly to the model performance as opposed to using averaged Hi-C signals across cell types. * indicates statistically significant differences using a paired t-test across the six cell types

As an alternative approach to our goal of integrating 5hmC enrichment with 3D chromatin organization, we next developed a deep learning method that uses a graphical convolutional network (GCN) architecture as developed by Bigness and colleagues [50] (Additional file 1: Fig. S3A). This GCN approach makes use of the GraphSAGE framework [55], which allows us to train an embedding-generator function in a cell-type, and then use this function in a previously unseen cell-type. We anticipated that, as long as the graphs and the node attributes (such as 5hmC enrichment and Input signal) are generated similarly for each sample, the trained function may retain predictive value across different cell types. Using our previously processed 5hmC, input and gene expression datasets, and integrating publicly available chromatin contact maps for six specific cell types (Additional file 2: Table S9; ones with matched Hi-C and 5hmC data), we trained our graphical 5hmC convolutional networks (“GhmCNs”) for the prediction task of gene expression status (Fig. 4A describes the model). We assessed the predictive ability of the developed models by unbiased metrics such as AUC and F1 scores, as we did previously.

Briefly, for each sample we built a graph based on the strongest Hi-C contacts per window, where the nodes are the 10 kb windows, and the edges are drawn between each window and its top 10 interactors. For each node, we obtained 5hmC and Input signal; if a node overlapped a gene’s TSS, that gene’s expression label (previously calculated) was assigned to the node (Methods). We trained all our GhmCN models based on reported hyperparameter tuning ( [50]; Methods). For each cell type, we collected and calculated the AUC score for the gene expression prediction task, based on the test set, and plotted the respective true positive versus the false positive rates. All the models we generated displayed an ability to discriminate between positive and negative cases, with all models showing AUC scores greater than 0.8 and four out of six with an AUC of 0.86 (Fig. 4B). Precision-recall curves for the same models also led to high AUPR values between 0.78 and 0.84 (Fig. 4B). To test the relevance of long-range interactions (or utility of Hi-C data in general), as well as to establish a baseline of our predictions, we regenerated our cell-specific GhmCN models by using only the 10 closest interactions to each bin/node (5 upstream and 5 downstream) (Fig. 4C). This provided a control for two well-known features: enrichment of enhancers in regions in the vicinity (1D genomic distance) of TSS and strong dependence of chromatin interactions on the same 1D distance. We observed that replacing 3D proximity (Hi-C) with 1D distance decreased AUC and AUPR for all our models (a statistically significant decrease when all six cell types are considered (Fig. 4C)), supporting the importance of cell-type specific long-range interaction information in making these gene expression predictions by linking key regulatory regions such as distal enhancers to gene promoters.

We also analyzed GhmCN predictions in comparison to the two ABC models discussed before. Note that the bin size in GhmCN models is 10 kb, and for ABC models, the bins are defined by ATAC-seq peaks; thereby, the two approaches work at different scales. By defining an overlap as an ABC region being fully contained in a GhmCN bin, we observed that more than half of both ABC-5hmC and ABC-H3K7ac regions were within GhmCN predictions, suggesting a level of consistency between all three models (Additional file 1: Fig. S3B–C). On the other hand, ABC-5hmC overlaps with over 11,245 of GhmCN predictions; this number is 5704 for ABC-H3K27ac. For both cases, however, a large number of GhmCN-specific regions remain but a comprehensive comparative analysis of such bins with ABC predictions, as we have done for ABC-5hmC versus ABC-H3K27ac, is challenging due to their coarse resolution (10 kb bins).

### GCN-based predictive models of gene expression from 5hmC are generalizable across cell types

One of the properties of these graphical convolutional networks is that they are not tied to a specific graph structure. In our study, the graph structure is composed of the Hi-C contacts (observed interactions between genomic regions); thus, the weights of a trained GhmCN model, generated by the input features of a specific cell-type (the graph structure, its associated 5hmC signal and input, and gene expression), can be used to process a different cell type’s input features and to make predictions. We tested the cross-cell type prediction ability of each of our models to assess the extent to which they are generalizable. We took the weights from the embedding-generating function of a model trained in a given cell type and assessed its predictive performance on each of the other cell types, using the new cell type’s input features (cross-cell type). We repeated this process on each of our 6 models. Additional file 1: Fig. S4A shows the cross-cell-type AUC scores, ranging from 0.81 when predicting gene expression in Activated B cells by using a model trained on resting B cells, to 0.54, when predicting gene expression in resting B cells using a model trained on Naïve CD4^+^ T cells (for the full set of results, see Additional file 1: Fig. S4B). Overall, we observed that the closer the cell type used in training to the one that is tested, the higher the predictive ability of the cross-cell type models, likely highlighting conserved features of 3D genome and Hi-C data across cells derived from a common progenitor. We corroborated this observation with the grouping pattern of the 6 cell types’ expression profiles through principal component analysis (Additional file 1: Fig. S4C).

Given our observations that the models trained in one cell type and tested in a different cell type depend on the similarity between the two cell types, we asked if we could use a combined set of Hi-C interactions to generate an aggregate model that could be used for predictions in previously unseen cell types with reasonable accuracy. To do this, we generated an aggregate (or averaged) 3D contact map, based on the known correlation of Hi-C contact frequencies and higher-order structures across cell types, largely determined by linear genomic distance [74, 75]. A similar approach of using an aggregate Hi-C signal has been employed by the ABC model [48]. Our motivation was that the use of an aggregate Hi-C map would benefit the analysis of cell types where maps of 3D contacts are not available.

To this end, we aggregated all the Hi-C datasets (Methods). Briefly, we down-sampled valid read pairs, merged them and normalized the resulting contact map, and reconstructed a graph that is then trained and tested one-by-one with each cell type’s 5hmC profile to obtain AUCs (Fig. 4D). The models for each cell type showed a better predictive performance with cell-specific contact maps rather than the averaged contact map (except equal AUC and AUPR for naïve CD8 T cells), a trend that is statistically significant for both AUC and AUPR values (Fig. 4E). The cell types that showed a noticeable drop in their AUC and AUPR scores when the aggregate Hi-C data was used were active and resting B cells which had the highest depth Hi-C maps with over 1 billion valid interactions (Additional file 2: Table S7). Overall, our results suggest that, while it is ideal to use cell-specific and sufficiently sequenced Hi-C contact maps, the averaged graph structure we generated can be used in conjunction with cell-specific 5hmC data to predict gene expression on cell types lacking available high-resolution Hi-C data.

For each cell type with a matching Hi-C and 5hmC enrichment profile, we repeated this Hi-C aggregation by holding out that cell type’s Hi-C data and then utilized the aggregated map with the held-out sample’s 5hmC profile for training and testing. We did not find any substantial difference between the average map containing all available Hi-C datasets and those with the sample of interest being held out in both AUC and AUPR scores (Table 5). These data support the robustness of our predictions in the absence of available Hi-C data from the cell type of interest, if Hi-C data from related cell types are available within the aggregated set. This is an important feature that may be useful in prioritization and target gene identification for enhancers that are characterized in rare cell types for which it remains challenging to generate chromosome conformation capture data.

**Table 5:**
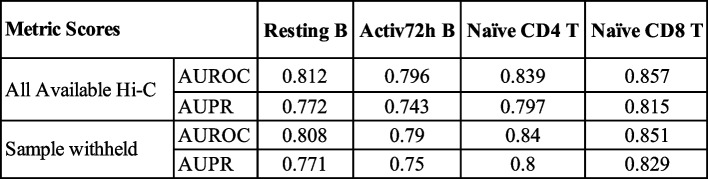
AUC and AUPR scores when either all samples were used or one sample was withheld from making the averaged contact maps

### Decoding the GCN models allows prioritization of putative enhancers with respect to their contribution to the prediction of target gene expression

We have integrated GNNExplainer with our GhmCN model to elucidate the model's predictive behavior. GNNExplainer, as proposed by [76], is designed to interpret the decisions made by graph-based neural networks by quantifying the contribution of edges and nodes to the prediction of a specific target node. In our study, this process involved several key steps to ensure a comprehensive understanding of the GhmCN model's predictive mechanisms, especially concerning gene expression predictions. Upon integrating GNNExplainer with GhmCN, the tool examines the prediction made by the model for a selected node, which in our context is a specific gene. GNNExplainer identifies the significance of the connections between the target node (a gene) and its neighbors (that can be a node with or without a gene TSS). The core of GNNExplainer's utility in our study lies in its ability to assign significance scores to each interaction between the node of interest and its adjacent nodes. These scores reflect the strength and importance of each interaction in contributing to the target node's predicted label, thus, allowing us to identify the most influential connections within the network.

Through our analysis and prioritization of nodes/regions that interact with gene-containing nodes, we found that a subset of the top ranked nodes for each gene contained regulatory elements with biological significance. This is depicted for two case studies where we focused on well-characterized loci harboring key genes for the cell type studied.

#### Case study A: prediction of putative enhancer regions for Aicda regulation in B cell activation

For further analysis, we focused on *Aicda*, which encodes AID (activation-induced cytidine deaminase), a crucial factor for class switch recombination (CSR). Recently [19], we reported two TET-dependent enhancers located ~10 kb (*TetE1*) and ~26 kb (*TetE2*) 5′ of the *Aicda* TSS, which both showed a progressive increase in 5hmC signal with time after stimulation with LPS and IL-4 to induce CSR. In both resting and activated B cells, these two experimentally validated regions were among the top 10 candidates reported by GNNExplainer, highlighting our model’s ability to capture putative functional enhancers (full set of top-10 nodes for *Aicda* in resting and activated B cells are listed in Additional file 2: Table S8). Among the other top-ranked interactions in activated B cells were the 10 kb window harboring the *Apobec1* TSS, as well as the region between *TetE2* and *TetE1*; all these regions are bound by known *Aicda* regulators [19].

**Fig. 5:**
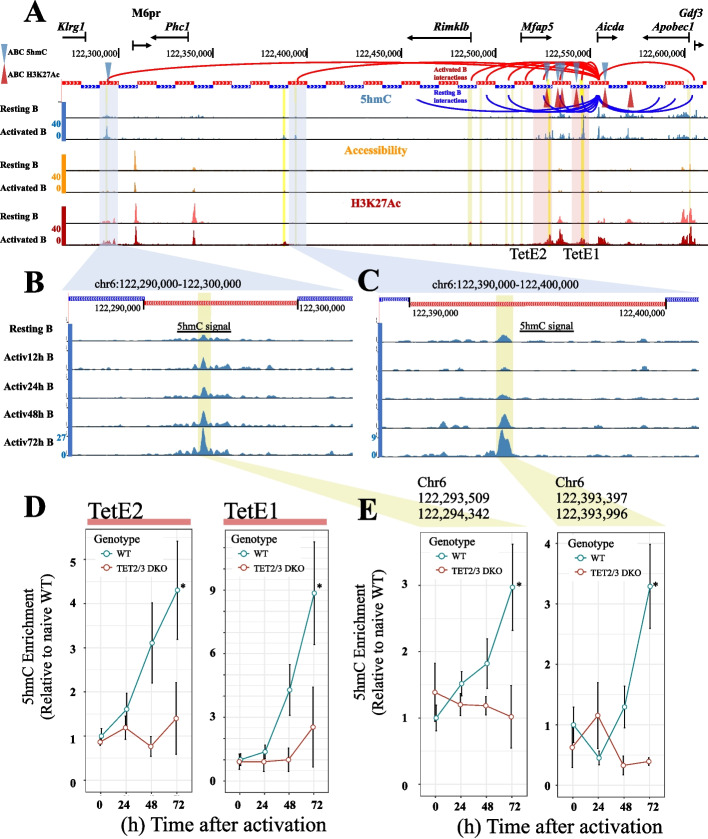
Novel regulatory regions prioritized in *Aicda* gene locus by GhmCN. **A** Genome browser overview of the GNNExplainer’s top interactions used to predict *Aicda* gene expression state in resting (green arcs) and activated (red arcs) B cells using GhmCN. Resting B cell interactions beyond the TSS of *Apobec* were omitted. Blue and red triangles indicate the ABC-predicted regulatory regions on activated B cells using ABC-5hmC (blue) or ABC-H3K27ac (red) models. Alternating red and blue thick lines indicate the 10-kb windows across the genome. Pink vertical highlights near the *Aicda* gene show the nodes containing the validated, TET-dependent *Aicda* enhancers “TetE1” and “TetE2.” The blue vertical highlights represent the two novel putative regions (260 kb and 160 kb away from *Aicda* promoter), which are predicted by GhmCN as important for predicting *Aicda* expression in activated but not in resting B cells. The ~ 260 kb away region is also predicted by our ABC-5hmC model but not by ABC-H3K27ac. **B**–**C** A zoom-in view of the 10 kb bins that are 260 kb (**B**) and 160 kb (**C**) away from *Aicda* TSS, respectively. The highlighted regions’ dynamic gain of 5hmC signal through B cell activation, a feature that is shared with the two previously validated *Aicda* enhancers, TetE1 and TetE2. **D** 5hmC-signal enrichment for TetE1 and TetE2 at 0, 24, 48, and 72 h after activation of WT (blue lines) and TET2/3 double knockout (DKO, red lines). **E** Similar plots for the two newly identified regions by GhmCN in active B cells. For (**D**) and (**E**), error bars represent the standard error of the mean, and * represents a Welch’s *t*-test *p*-value < 0.1 as published in [19]

Notably, we also observed two long-distance interactions, more than 100 kb away from the *Aicda* TSS, that were prioritized by GNNExplainer in activated but not resting B cells. These two intergenic regions were located ~260 kb and ~160 kb 5′ of the *Aicda* TSS (Fig. 5A, Additional file 2: Table S8, 1^st^ and 2^nd^ row, respectively), and have not previously been reported to have regulatory roles in *Aicda* expression. We explored 5hmC distribution and the dynamics of 5hmC enrichment within these 10-kb windows (Fig. 5B–D) using 5hmC mapping data (by CMS-IP) obtained from WT and double Tet2/3-deficient B cells, resting or activated (stimulated) for 24, 46, and 72 h with LPS and IL-4 [19]. A region inside each node significantly gained (*p*-value < 0.1) 5hmC signal after 72 h of stimulation (chr6:122,293,509–122,294,342 and chr6:122,393,397–122,393,996, respectively), a pattern reminiscent of the 5hmC gain observed in the known Tet-dependent *Aicda* regulators TetE2 and TetE1 [19] (Fig. 5D–E).

Taken together, the top-ranked interacting regions identified by GNNExplainer highlight the validated *Aicda* enhancers *TetE2* and *TetE1* and predict two novel distal regions that also have the features of *bona fide Aicda* enhancers, in that they gain 5hmC after stimulation in a manner similar to *TetE2* and *TetE1*. Importantly, although *TetE2* and *TetE1* were also identified by both ABC-5hmC and ABC-H3K27ac models, the ~260-kb region with a strong 5hmC signal was identified by our ABC-5hmC model but missed by ABC-H3K27ac. The ~160-kb region, on the other hand, was missed by both ABC models. In light of these results, experimental validation of these new regions as *de novo Aicda* enhancers, possibility in the context of simultaneous perturbations to *TetE2* and *TetE1*, in B cells both in culture and *in vivo*, is needed to fully understand their functional role in regulation of *Aicda* gene expression during B cell activation. Potentially, they may be involved in setting up the *Aicda* locus in developing B cells for future transcription in mature B cells, rather than directly regulating *Aicda* transcription in mature B cells after stimulation.

#### Case study B: prediction of putative enhancer regions for *Il4* in Th2 cells

Type 2 helper T (Th) cells (Th2 cells) are generated by polarization of naïve CD4^+^ T cells in the presence of interleukin (IL)-4, a potent inducer that directs differentiation of naïve CD4^+^ T cells into CD4^+^ Th2 effector cells [77]. Many studies have focused on *Il4* gene regulatory networks: key regions within the last exons of *Rad50 * [78, 79], a gene located 5′ of *Il4*; conserved non-coding sequence 2 (*CNS2*) located between the TTS of *Il4* and *Kif3a * [80]; and *CNS1* in the intergenic space between *Il4* and *Il13* [81, 82] have been reported as *Il4* enhancers [83]. *CNS1* is essentially fully methylated (5mC + 5hmC) in WT naïve CD4^+^ T cells and becomes substantially demethylated during Th2 differentiation, whereas *CNS2* is poorly methylated (5mC + 5hmC) in naïve T cells and remains demethylated in differentiated Th2 cells [84].

**Fig. 6:**
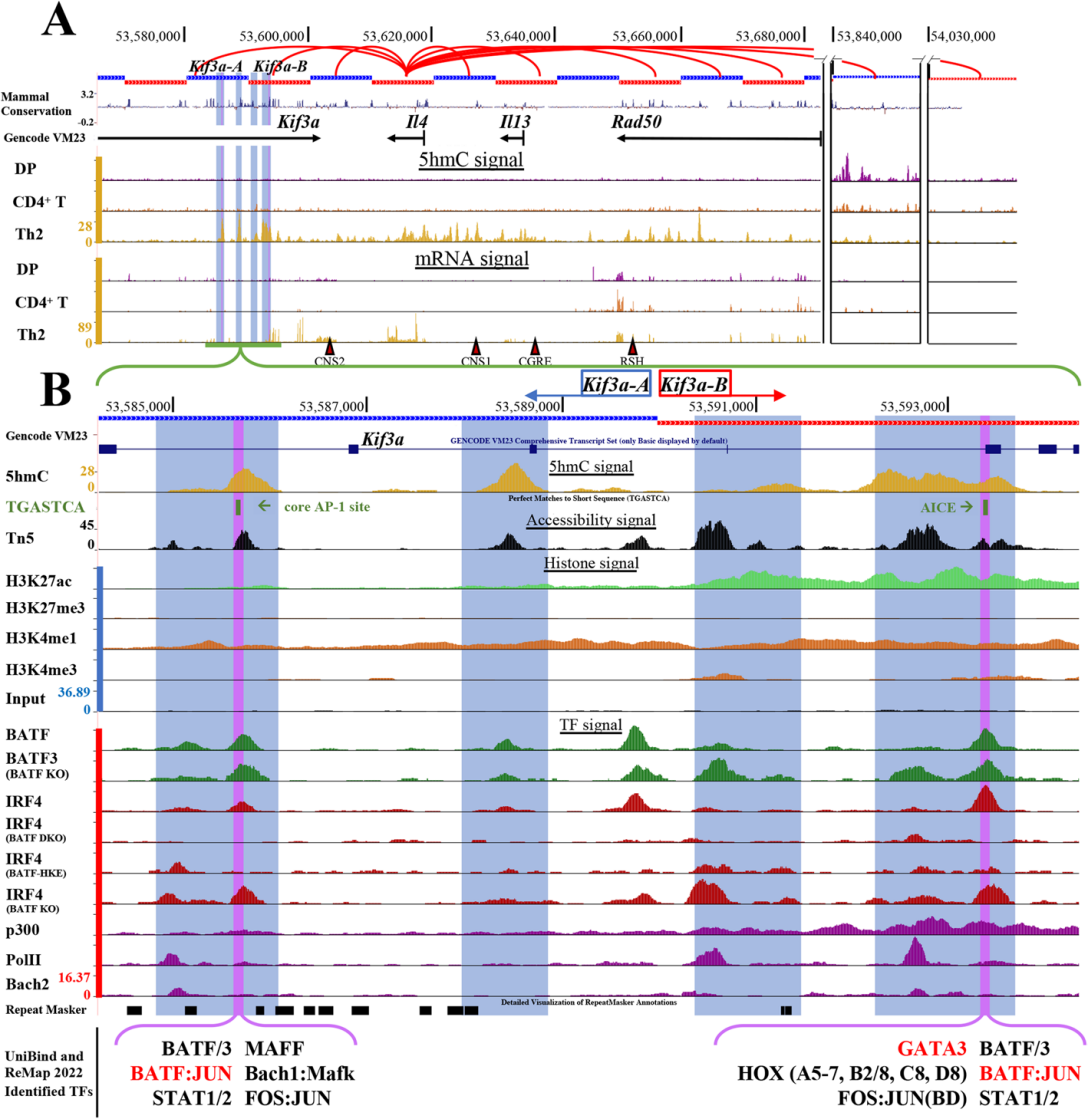
Regulatory regions identified in Il4 locus from Th2 cells. **A** Genome browser overview of the GNNExplainer’s top interactions used to predict *Il4* gene expression in Th2 cells (red arcs). (Top) Alternating red and blue thick lines indicate the 10-kb windows across the genome. For visualization, two stretches between the 10-kb window containing the *Il4* gene and two interacting 10-kb windows 5′ of the *Rad50* gene (right side of the panel) were omitted. (Middle) 5hmC signal tracks from DP, CD4 T naïve, and Th2 cells, followed by (Bottom) RNA-seq signal in the same cells, illustrating Th2-specific activity as expected. The green segment (shown as a zoomed view in B) represents two *Il4*-interacting nodes (here termed Kif-A and Kif-B) that have not yet been tested for roles in *Il4* gene regulation. **B** A zoomed-in browser view shows that both *Kif3a-A* and *Kif3a-B* regions harbor multiple 5hmC signal peaks with one in each region containing a perfect match to the AP1–IRF composite element (AICE) sequence motif (TGASTCA) that binds BATF and IRF4. Purple highlights represent 5hmC peaks with AICEs that also had co-binding of BATF and IRF4, and whose IRF4 binding is lost in BATF DKOs and HKE (a triple mutant form of BATF that suppresses IRF4 interaction). These regions also show strong signals of accessibility and some level of active histone marks such as H3K27ac and H3K4me1 in addition to binding of a group of TFs identified using UniBind and ReMap2022 databases (bold text at the bottom)

Among the top 10 interactions associated to the *Il4* TSS, 4 contained reported regulatory regions (Fig. 6A): (i) *CNS2*, also known as hypersensitive site V (chr11:53600000:53610000) [80, 85, 86], (ii) *CNS1*, located between *Il4* and *Il13* (chr11:53620000:53630000) [80–82, 87], (iii) *CGRE*, 1.6 kbp upstream of *Il13* (chr11:53630000:53640000) [80, 88], (iv) *RHS6/7* and *RHS5*, located in the last exon of the *Rad50* gene (chr11:53650000:53660000) [78, 79, 89]. Of the other interactions, two (here termed *Kif3a-A* and *Kif3a-B* for convenience) appeared particularly relevant based on their proximity to the *Il4* gene; none of the other T cell samples (DP, CD4^+^, and CD8^+^ naïve T cells) had these two regions in their top interactions (Additional file 2: Table S11, see regions demarked by the black box). At the *Kif3a-A* and *Kif3a-B* regions, we observed clear 5hmC signal peaks and strong presence of transcription factor binding sites (TFBS) found by Remap2022 [90], UniBind [91], and analysis of public ChIP-seq datasets within chr11:53580000–53600000 (Fig. 6B), including for Foxo1, NFAT1, 2 and 4, CREB, STAT, MYC, Fos, JunD /B, BATF, MAFF, IRF4 and additional basic region-leucine zipper (bZip)-related transcription factors. Although a previous study [92] showed that inhibition of Foxo1 had no effect on *Il4* expression, several reports have shown evidence of the crucial role of NFAT, IRF4, BATF, and other bZIP factors in Th2 cell generation and *Il4* expression in both mouse and human cells [93–95].

To explore the potential roles of the *Kif3a-A* and *Kif3a-B* regions in regulating *Il4* expression, we downloaded accessibility data, chromatin immunoprecipitation (ChIP-seq) data for multiple epigenetic marks, as well as ChIP-seq data for several transcription factors (Additional file 2: Table S11). Within the 5hmC peaks, each of these two nodes (Fig. 6B, pink highlights) displayed strong co-binding of key transcription factors such as BATF and IRF4, and IRF4 binding was lost in BATF KO and BATF/BATF3 DKO Th2 cells. The *Kif3a-A* and *Kif3a-B* regions were accessible and displayed H3K27ac enrichment in Th2 cells, *Kif3a-A* contained one perfect match (chr11:53585651–53585753) to the activating protein 1 (AP-1) binding consensus sequence (TGASTCA), and *Kif3a-B* (chr11:53593319–53593416) a very close match to the AP-1–IRF composite elements (AICE2; TacCnnnnTGASTCA), known to enable IRF4/8-dependent transcription by cooperative binding with BATF, resulting in expression of genes associated with activation and differentiation for Th2, Th17, B, and dendritic cells [96, 97]. Kuwahara and colleagues [93] showed that there is a positive feed-forward (amplification) loop between *Il4* and *Batf* to induce Th2 cell differentiation, where the BATF:IRF4 complex is key for IL-4 expression, and overexpression of IL-4 further augments BATF expression. Both ReMap 2022 and UniBind provided further evidence for BATF and IRF4 binding as well as general bZIP TF binding in *Kif3a-A* and *Kif3a-B*. We therefore speculate that the *Kif3a-A* and *Kif3a-B* regions are unreported *Il4* enhancers mediated through bZIP TF family members, such as the BATF:IRF4 complex. This hypothesis warrants further functional investigation.

## Discussion

5hmC signal enrichment has previously been associated with positive gene expression, enrichment of the H3K4me3 mark, and RNA polymerase II [98]. Here, we explored this association further by employing a fully connected deep neural network (FCDNN) that models signals from cell type-specific 5hmC enrichment to predict gene expression. We also showed that by integrating the 5hmC signal with 3D chromatin structure (as obtained by Hi-C-derived genome-wide contact maps) using graph neural networks, and obtaining feature importance scores from the trained models, we can identify distal regions containing known and novel regulatory elements, e.g., enhancers) for important genes in immune cells. In addition, we demonstrated the feasibility of using aggregated Hi-C data from related cell types to reliably predict gene expression and to explain the contributions of different distal enhancers to these predictions. To our knowledge, this work is the first systematic approach to gene expression prediction and enhancer prioritization using a 5hmC signal.

On the FCDNN modeling, when we calculated the AUC in models trained and tested on the same cell type, we obtained a median AUC of 0.89 across 49 samples. Compared to other machine learning models we used as baseline (SVM, random forest, and logistic regression), FCDNN showed improved predictive performance consistent across different settings. Although previously developed methods that use multiple histone marks, and complex network architectures such as kernels and convolutions in DeepChrome [38], and a hierarchy of multiple Long Short-Term Memory modules with recurrent and memory cells in AttentiveChrome [39] achieved AUCs around 0.8, these models were only trained and tested on the same cell-type. Here, we wanted to assess whether our predictive models would generalize to unseen cell types. For this, we first developed what we call combined models that utilize data from all samples for training while leaving out entire chromosomes for validation and testing. These models showed a promising predictive power with an overall AUC of 0.87 and were robust to the choice of chromosomes held out from training. We next generated similar combined models but by completely leaving out 10 samples from the training and also leaving out entire chromosomes to avoid effective memorization. Obtaining an AUC of 0.86 across all unseen cell types from this model showed that our models are generalizable. These results suggest that generalized features of 5hmC patterns associated with gene expression can be obtained using deep learning and utilized for predicting gene expression in samples/cell types that are unseen or do not have gene expression measurements (e.g., samples with degraded RNA). Another important finding from our DNN models was that the bins with the greatest contribution to gene expression prediction were found at the immediate downstream region of the TSS (~500bp), a region that is excluded from Hi-C analyses. Whether this observation is related to previously characterized downstream promoter elements (DPEs) or the interplay of methylation/demethylation with TF binding events in the broader downstream region remains to be explored.

In this work, we also developed two novel approaches to utilize 5hmC enrichment together with 3D chromatin organization information to better understand distal gene regulation. In our adaptation of the ABC model, we used 5hmC as Activity (a result of TET enzymatic activity itself) rather than the H3K27ac signal to compare and contrast the prioritized enhancer regions and their characteristics. Our findings suggest that the 5hmC signal in ABC allows us to capture a very large fraction of regions that are found by the standard ABC approach that uses H3K27ac. In addition, ABC-5hmC captured thousands of new regions that are distal to promoters and in addition to 5hmC enrichment have weaker but enriched ATAC-seq signals (as expected since we start with ATAC-seq peaks). The biological significance of these regions needs to be tested using functional genomics approaches in order to understand whether or what roles they play in distal gene regulation.

In our GhmCN machine learning models, we used a 3D chromatin structure to connect gene expression to 5hmC signal levels (10 kb bins) using the top interacting regions for each gene. By doing this, we integrated the distal regulatory regions and their 5hmC signal distribution to obtain cell-specific models of gene expression. When we tested cross-cell-type predictions, the accuracy dropped proportional to the distance between the cell types used for training and testing. However, when we generated an averaged Hi-C interaction map from subsampled multiple Hi-C datasets (cell types included naïve and activated B cells; DP and CD4^+^ naïve T cells; CD8^+^ naïve, effector and exhausted T cells; LSK, Th2, and BMDMs), we showed that these models conserved strong predictive ability for unseen genes and also unseen cell types (i.e., Hi-C data of the cell type withheld from Hi-C aggregation). This provided evidence that cell-type-specific 5hmC enrichment signals can be a powerful way to predict gene expression when integrated with averaged 3D chromatin structure data. However, our comparison utilizing a cell-specific Hi-C matrix versus aggregate Hi-C data demonstrated a drop in predictive performance for cells with deeply sequenced Hi-C data (e.g., resting and activated B cells). This suggests that the loss or dilution of cell-specific looping information, likely involving distal regulatory regions, may be responsible for lower predictive performance; hence, utilizing information about cell type-specific regulatory regions may be critical at least for a subset of genes. To further understand the nodes (regions) and edges (Hi-C interactions) that are learned as predictive in our GhmCN models, we used GNNExplainer, a tool that assigns relative importance to each edge and node feature in a graph. This analysis proved to be a useful way to identify the putative regulatory regions among those interacting with a gene (i.e., regions that are most important in predicting expression).

Comparing our results with published work, we found that the top candidates (genomic regions) for regulating exemplar genes were consistent with observed roles associated to those regions. For instance, the *TetE1*- and *TetE2*-containing nodes (harboring two distinct validated enhancers) were ranked in the top 5 most important interactions in activated B cells by GhmCN and were also captured by ABC models. Moreover, our prioritization of the candidate regions with respect to GNNExplainer scores allowed us to identify novel regions with potential enhancer activity, which have yet to be validated. We believe the two approaches we developed here for the utilization of 5hmC and Hi-C data will be of value for prioritizing putative functional enhancers that are missed by an H3K27ac-centric approach to enhancer discovery and enhancer-promoter linkage.

There are some technical and some conceptual limitations to our work as it is presented here. For instance, while Hi-C and 5hmC signal enrichment constitute a powerful pair, Hi-C is substantially more expensive and has lower resolution compared to 5hmC. Our results showing that an averaged Hi-C contact map from an ensemble of cell types provides reasonable predictions addresses, to an extent, the situation when Hi-C data is not available but 5hmC is. However, both Hi-C and 5hmC measurements can benefit from higher resolution methods. All of the 5hmC data we utilized in this work are from immunoprecipitation-based assays (e.g., CMS-IP, hMeDIP, hMeSeal) for the identification of 5hmC-enriched regions (peaks). Single base resolution information, such as those from recently developed six-letter-seq [99], will likely enable finer-scale mapping of regulatory elements impacting gene regulation. On the Hi-C side, broader adoption of the latest techniques such as Micro-C [100], Micro Capture-C [101], and Region Capture Micro-C [102] may provide deeper contact maps required to fill the resolution gap. Another potential limitation to our approach is the dependence of the 5hmC signal on CpG content. Enhancers that are CpG-poor, even if highly active, might not display detectable/strong 5hmC enrichment, and therefore would be missed by 5hmC-based approaches such as ours.

As a future direction, it would be interesting to eliminate the use of Hi-C and to be able to link 5hmC-enriched enhancers to their target genes solely from 5hmC measurements. Given the dynamic nature of 5hmC deposition at newly utilized enhancers [19], this would require surveying enough differentiation steps or time points with gene expression and 5hmC measurements to derive correlations. Another important application of our approach could be for utilizing 5hmC distribution in cell-free (circulating) DNA, which can be used to detect cell-type-specific features such as genes predicted to be highly expressed by our model that are markers of specific cell types or can point to tissue of origin. Our approach would also be useful when the only source of cellular material is DNA, or if cells are subjected to processes that compromise their viability, such as formalin-fixed paraffin-embedded (FFPE) preserved samples, for which it is not possible to obtain information about gene expression since RNA cannot be extracted. Since 5hmC is a stable, covalent DNA modification that survives DNA extraction protocols, assessing 5hmC signals would enable the study of such samples and would also provide estimates of differences in gene expression across different conditions (e.g., stimulated vs unstimulated cells, healthy vs tumor tissue). Given the enrichment of 5hmC in enhancers, and our demonstration that using aggregate contact maps from other relevant cell types is a reasonable approach, 5hmC (CMS-pulldown) measurements alone may be sufficient to provide a glimpse of epigenetic regulation in such samples. Exploration of potential distal regulatory elements and chromatin contacts for such samples would not otherwise be possible. Our study sets the stage for future work that utilizes 5hmC, on its own or in addition to other genomics and epigenomics datasets, for modeling gene regulation.

## Conclusions

Our study sets the stage for future work that utilizes 5hmC distribution genome-wide for modeling gene regulation. The approaches developed here, either utilizing 5hmC enrichment on its own or together with 3D chromatin organization, show that 5hmC distribution in proximal and distal regulatory elements is informative of gene expression and allows prioritization of putative functional enhancers that are missed by previous approaches. Whether 5hmC plays a direct role in distal gene regulation remains to be tested using functional genomics approaches.

## Methods

### Compilation of 5hmC and gene expression datasets

We downloaded 5hmC-immunoprecipitation sequencing datasets, generated using multiple different techniques (CMS-IP-seq, hMEDIP, HMCP, GLIB-seq, and hMe-Seal) for 153 samples representing 40 different cell types from the published literature; as well as RNA-seq from the same cell types (Additional file 2: Table S1, Additional file 2: Table S2 and Additional file 2: Table S3 contain the GEO IDs and replicate information for all samples analyzed). In Additional file 2: Table S4 we show the triad of 5hmC enrichment, corresponding 5hmC input, and matched gene expression profile for each cell type.

### Alignment and uniform processing of 5hmC datasets

All 5hmC sequencing experiments were processed with the same pipeline as follows. We downloaded the raw reads and mapped them to the mm10 genome reference assembly using Bsmap [103]. Unmapped reads were remapped after using TrimGalore [104] and added to the mapping results after both files were sorted with SAMtools [105]. PCR duplicates were identified and removed using Picard Toolkit’s *MarkDuplicates* function (Broad Institute. Picard Toolkit 2018). Mapping results aligned to ENCODE’s blacklisted regions [106] were removed before further analysis. We generated HOMER’s *TagDirectories* followed by HOMER’s *makeMultiWig* tracks for visualization in the genome browser [107]. The 5hmC (and input) signal in the graph’s nodes was obtained using GenomicAlignments’s *summarizeOverlaps* function [108].

### Quality control and representative replicate selection for 5hmC data

We executed QC metrics to remove low quality samples from the data compendium (i.e., location of the highest and lowest signal window, signal ratio between highest and lowest points, and clean signal among low and high labeled genes). Each sample’s 5hmC data replicates that are inconsistent with others or have patterns of low 5hmC enrichment/depletion were discarded (112 out of 153 replicates passed QC). We further filtered out datasets to only include one replicate that passed QC metrics for each cell/sample type (randomly chosen) to avoid data leakage (49 replicates out of the 112 samples passing QC).

### Alignment and uniform processing of RNA-seq datasets

All gene expression data was processed using a STAR aligner [109]. We downloaded the raw reads and mapped them to the UCSC genome annotation database for the Dec. 2011 (GRCm38/mm10) assembly of the mouse genome. Counts per gene were obtained using *FeatureCounts* [110]. Identical results were obtained when using STAR’s count algorithm.

### Extraction of 5hmC features and expression labels for each gene

For each sample, 5hmC enrichment and the 5hmC input signal were processed together to produce the inputs for our proposed models. To determine the set of genes to be used, we utilized UCSC gene annotations for the Dec. 2011 (GRCm38/mm10) assembly of the mouse genome and excluded genes with sizes smaller than 1 kb leaving us with 21,752 genes. Data from RNA-seq experiments were then used to define labels for each of these remaining genes using the median TPM value for that sample as a threshold to label genes as either “high” (above median) or “low” (below median) expression (Additional file 1: Fig. S1C). For each gene longer than 1 kb, we extended the promoter both upstream and downstream by 5 kb, and divided these 10 kb stretches into 100 equally sized bins (100 bp per bin). We also took 1.5-kb regions both upstream of the TSS and downstream of the TTS, resulting in 15 equally sized 100-bp bins for each gene. We also split the gene body (from TSS to TTS) into 100 variable-sized bins to account for varying gene lengths. We used this set of 230 bins per gene to obtain the raw 5hmC signal from the mapping results and proceeded to RPKM-normalization based on the sequencing depth per sample and then performed a bin signal normalization. (Additional file 1: Fig. S1A–B).

### Analysis of ChIP-seq datasets

All downloaded ChIP-seq data was processed similarly to the 5hmC enrichment datasets with the only difference being the use of BWA mem [111] as opposed to Bsmap for the mapping steps.

### Analysis of ATAC-seq datasets

Paired raw reads were aligned to the Mus musculus genome (mm10) using Bowtie [112]. Unmapped reads were trimmed to remove adapter sequences and clipped by one base pair with TrimGalore [104] before being aligned again. Sorted alignments from the first and second alignments were merged together with SAMtools [105], followed by the removal of reads aligned to the mitochondrial genome. Duplicated reads were removed with Picard Toolkit’s *MarkDuplicates* (Broad Institute. Picard Toolkit 2018). Reads aligning to the blacklisted regions [106] were removed using bedtools intersect [113]. Final mapping results were processed using HOMER’s *makeTagDirectory* program followed by the *makeMultiWigHub* program [107] to produce normalized bigWig genome browser tracks.

### Alignment and uniform processing of Hi-C datasets

All datasets were processed using HiCPro [114]. We downloaded the raw reads and mapped them to the UCSC genome annotation database for the Dec. 2011 (GRCm38/mm10) assembly of the mouse genome. We obtained the appropriate restriction enzyme per sample from their corresponding manuscript’s published methods, required for HiCPro’s configuration file. For samples with either multiple lanes or multiple replicates, we generated a merged sample folder and re-computed the ICE [115] normalized matrices by running HiCPro and the steps “-s merge_persample -s build_contact_maps -s ice_norm.” For all analyses in this work, we used 10 kb resolution bins for Hi-C data.

### Traditional Machine Learning methods

All three methods implemented as baseline, logistic regression, random forest, and support vector machines, were run with default parameters in R (version 3.3.3), from packages “tibble”, “randomForest” and “e1071” respectively, using all the 230 bins as the explanatory variable and the gene expression state as the target. The Validation and Test datasets per sample consist of the genes in chr5 and chr4, respectively. Training was performed using the remaining chromosomes. For the AUC scores, we used the library pROC’s *roc* function. Wilcoxon signed-rank test with continuity correction was used to compare the AUC score distributions between different predictive models.

### Majority vote baseline

As another baseline method, we developed a simple method that utilizes the majority vote of low vs high label of a gene (and hence allowed to memorize gene expression labels from training samples) across all training samples to predict the same gene’s expression in one held-out sample. For a given gene, and an excluded sample, the baseline label was assigned as the label that was present in more than 24 samples (i.e., more than half of 48 training samples after holding out one sample for testing).

### Promoter CpG content differences for genes from different expression categories

To investigate the relation between CpG content in the promoter (defined as +/− 1 kb around the TSS) and expression, we first categorized the genes into 5 major (partially overlapping) groups according to their expression status and expression variability in the 49 samples analyzed: (1) ubiquitously expressed genes obtained across 17 mouse tissues [116] (provided as a table under dataset 1 in the original publication); (2) genes that were always “High” across our 49 samples; (3) Genes that were always “low” across our 49 samples; (4) variable genes defined as the set of genes whose underrepresented label covered at least a third of the samples; (5) genes with zero expression (TPM = 0) across all samples. We note that this categorization leaves out a portion of genes that have variable gene expression labels. For gene promoters in each of the groups mentioned, DNA sequence was fetched using the Dec. 2011 (GRCm38/mm10) assembly of the mouse genome and CpG content was calculated using pybedtools and bedtools [113, 117].

### Deep neural networks

We developed our DNN models in pyTorch and translated them into Keras for the DeepLift analysis. After hyperparameter tuning with the validation dataset, we trained our single-cell models using the following hyperparameters: hidden layers = 3, neurons per layer (L#): L1 = 200 (input to hidden), L2 = 100 (hidden), L3 = 50 (hidden), L4 = 1 (output), learning rate = 0.0001, probability of dropout in hidden layers = 0.15, total epochs (*e* = 40) and minibatch size of 128 samples. For the Combined model we increased to 60 the number of epochs. We aimed at having a similar number of genes in the test and development datasets, therefore we used chr5 genes as our validation dataset (*n* = 1340 genes) and chr4 genes as our test dataset (*n* = 1316 genes). The training dataset was composed of the remaining chromosomes (*n* = 19,042 genes). For the analysis assessing the robustness of our combined model (Fig. 1D), leaving out a different set of chromosomes for testing, we generated an array of 19 entries and two chromosomes each time where no chromosome would appear twice as either test or validation. We then trained 19 different models using these combinations and reported the AUC scores on the unseen, test dataset. To avoid effective memorization of average values by our models, a pitfall highlighted in gene expression prediction tasks [56], for combined models, we withheld the same set of genes from each cell type, hence, leading to a truly unseen dataset for accurate calculation of predictive performance.

### DeepLift activation logic

We took the minimum and maximum observed feature values as a range to survey across to obtain a float such that when used across all bins, the neural network output layer will not return either 0 or 1 (i.e., 0.49, not specific for High or Low expressed genes). We used these values as our “neutral reference” to decode the trained network using as input the test dataset. The decoding was performed twice, once for the observed High genes and once for the observed Low genes. For learning feature importance, we used DeepLift with a target layer index (− 2), which computes explanations with respect to the logits. The score layer index we used was (0) which correspond to the scores for the input layer. Each input feature (230 bins) will have a score per sample used to decode the network. The plots shown (Fig. 2B–C) represent the mean score plus/minus the standard deviation per bin.

### Graph convolution networks

We employed the same strategy as reported by Bigness and colleagues [50]. Briefly, we followed the GraphSAGE framework [55] formulation as the structure for our GCNs due to its portability and lack of restriction to a specific graph structure. The window size we used to capture both 5hmC signal enrichment and input (control) and used in the convolution embeddings was 10 kb, a single measure per node. The model layers consisted of a series of convolutions (convolutions = 2) interconnected by a ReLU operational unit, followed by a multi-layered perceptron of three layers with a 50% dropout chance to avoid overfitting. In our methodology, we started by normalizing the Hi-C signal using the ICE algorithm (115). To further refine this normalized data, we implemented a distance normalization by deducting the median values of the upper diagonals from each data point (negative values are set to zero). Subsequently, we constructed a network model per chromosome wherein each node is connected to its top-10 nearest neighbors, denoted by *k* = 10. Due to the undirected nature of the network, certain nodes may be connected to more than ten neighbors. This is because a single gene node may rank within the top 10 neighbors for multiple other genes. It is important to note that we experimented with a network of 15 neighbors per node. However, we encountered issues with memory usage, a challenge also highlighted by [50]. To assign genes to the nodes, we used as anchor point the gene’s TSS coordinates. When a node had more than one TSS (overlapping genes), the mean expression was taken for node label assignment. A gene was marked as either being “high” or “low” based on the median gene expression of the sample, as described before. Training the network made use of a mask to consider only the nodes with at least one TSS (to ensure a valid prediction could be made) and by using three convolution layers we indirectly set the number of k-hops to 3 (up to three interactions away are convoluted over and integrated for the prediction). The train, validation, and test fold datasets per sample were split into 70/15/15% from the total.

### GNNExplainer analysis

GNNExplainer, a framework for interpreting Graph Neural Network predictions, was employed to elucidate the contributions of node interactions within our GhmCN model. We utilized the GNNExplainer function from the torch_geometric.explain library using default parameters and the suggested number of 200 epochs for node-level explanations. We visualize the generated output using the EGA_visualize_subgraph function, which plots the target node together with its prioritized neighbors with edge color (darkness) indicating the order with respect to their significance scores. We explained the queried nodes up to 1-hop away (k-hops = 1).

### Hi-C dataset aggregation

We down-sampled all Hi-C datasets to a total of 183M randomly selected valid interactions (Additional file 2: Table S9; DP and Th2 cells were excluded due to low coverage) and obtained a combined Hi-C contact map as a new graph structure. This contact map was then normalized using the iterative correction (ICE) technique [115], further normalized by distance when preparing the GhmCNs. The normalized genomic interactions were used to generate a GhmCN of each cell type’s 5hmC profile as described above.

### ABC modeling

H3K27ac (ChIP-seq) and ATAC-seq data were processed as indicated above. The HiC-Pro’s ICE-normalized interaction matrices were transformed to a bedpe format and gzipped. We used Dec. 2011 (GRCm38/mm10) annotation to define the gene TSS positions. The mouse-blacklisted regions were downloaded from https://github.com/Boyle-Lab/Blacklist/blob/master/lists/mm10-blacklist.v2.bed.gz. BigWig tracks were generated using “bamCoverage” from deeptools [118]. We called peaks for 5hmC, H3K27ac, and ATAC-seq accessibility signal using MACS2 [119] calling summits and a *p*-value of 0.1. The HiC-Pro ICE-normalized data was transformed to bedpe format and separated by chromosome, required to run the ABC model. The code used to run ABC is provided in our Github and available in the zenodo archive under Availability of Data and Materials. These datasets are the input required to run the Activity-by-Contact enhancer prediction tool’s functions, which we used as follows: we ran src/makeCandidateRegions.py with parameters --peakExtendFromSummit 250 --nStrongestPeaks 150000; continued by src/run.neighborhoods.py with default parameters; Followed by src/predict.py with parameters --hic_resolution 10000 --scale_hic_using_powerlaw --threshold .02 --make_all_putative. The remaining parameters were either the required input files or defaulted. We ran ABC with 5hmC as the activity signal indicator and compared it to using H3K27ac (ABC-5hmC vs ABC-H3K27ac).

### Venn Diagrams and Heatmaps of regions from different predictive models

Overlap of regions predicted by ABC-H3K27ac and ABC-5hmC models was defined by using bedtools intersect with -u option [113]. Regions unique to each method are identified using bedtools intersect with -v option. These BED regions were then given as input to deeptools’ computeMatrix function followed by plotHeatmap [118]. The Venn diagrams were plotted using Python’s “matplotlib_venn” and pyplot function from matplotlib [120]. For overlap calculations with GhmCN predicted regions (10 kb bins), we checked whether the ABC-predicted region was within the GhmCN bin.

### Supplementary Information

**Additional file 1:** Figures S1-S4. Supplementary Figures with their captions.

**Additional file 2:** Table S1-S11. Supplementary Tables with their descriptions and captions.

**Additional file 3:** Review history.

## Data Availability

All data and code used for this study are publicly available. Table S1, Table S2, and Table S3 under Additional file 2 contain the GEO project ID, sequencing technique, PubMedID, and citation reference for 5hmC immunoprecipitation, and gene expression profiles. An example dataset to test the GhmCN network is available through the Zenodo archive at [121]. The version of the open-source software developed in this work is also available through Zenodo [122] with Creative Commons License CC BY-NC-SA 4.0. All (other) data needed to evaluate the conclusions in the paper are present in the paper or provided in the Additional files.
