# Supplementary material for: Predicting gene expression state and prioritizing putative enhancers using 5hmC signal
Source: Genome Biol. 2024 Jun 3;25:142. doi: 10.1186/s13059-024-03273-z (PMC11145787; doi:10.1186/s13059-024-03273-z)
Supplement: Supplementary file 1 — Additional file 1: Figures S1-S4. Supplementary Figures with their captions. [file 13059_2024_3273_MOESM1_ESM.pdf]

Fig. S1

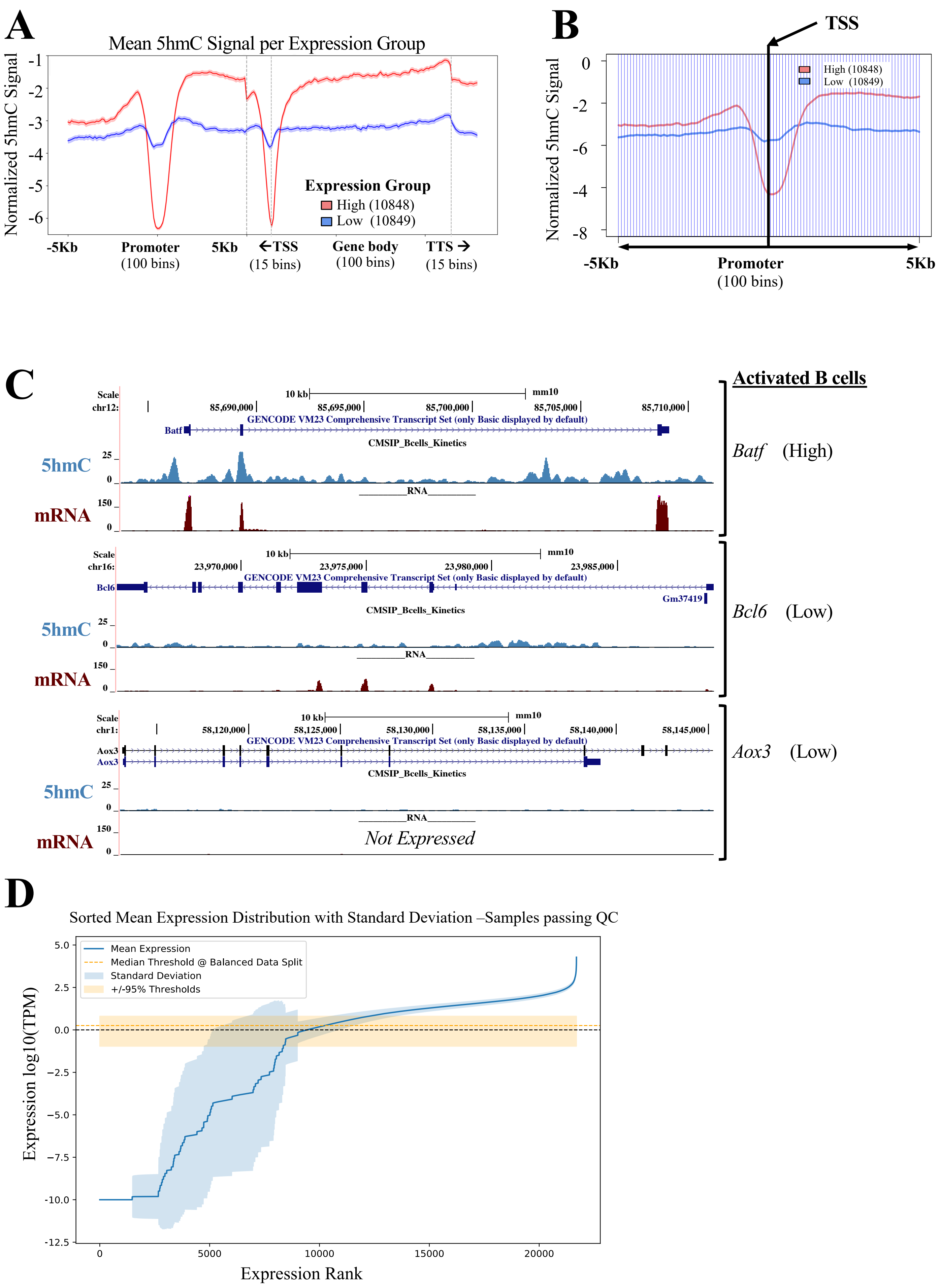
A
Mean 5hmC Signal per Expression Group
Normalized 5hmC Signal
-1
-2
-3
-4
-5
-6
Expression Group
High (10848)
Low (10849)
-5Kb
Promoter
(100 bins)
5Kb
←TSS
(15 bins)
Gene body
(100 bins)
TTS →
(15 bins)
B
TSS
Normalized 5hmC Signal
0
-2
-6
-4
-8
High (10848)
Low (10849)
-5Kb
Promoter
(100 bins)
5Kb
C
Activated B cells
Scale
chr12:
10 kb
mm10
85,690,000
85,695,000
85,700,000
85,705,000
85,710,000
GENCODE VM23 Comprehensive Transcript Set (only Basic displayed by default)
Batf
CMSIP_Bcells_Kinetics
5hmC
25
0
RNA
mRNA
150
0
Batf (High)
Scale
chr16:
10 kb
mm10
23,970,000
23,975,000
23,980,000
23,985,000
GENCODE VM23 Comprehensive Transcript Set (only Basic displayed by default)
Bcl6
Gm37419
CMSIP_Bcells_Kinetics
5hmC
25
0
RNA
mRNA
150
0
Bcl6 (Low)
Scale
chr1:
10 kb
mm10
58,120,000
58,125,000
58,130,000
58,135,000
58,140,000
58,145,000
GENCODE VM23 Comprehensive Transcript Set (only Basic displayed by default)
Aox3
Aox3
CMSIP_Bcells_Kinetics
5hmC
25
0
RNA
mRNA
150
0
Not Expressed
Aox3 (Low)
D
Sorted Mean Expression Distribution with Standard Deviation –Samples passing QC
Mean Expression
Median Threshold @ Balanced Data Split
Standard Deviation
+/-95% Thresholds
Expression log10(TPM)
5.0
2.5
0.0
−2.5
−5.0
−7.5
-10.0
-12.5
0
5000
10000
15000
20000
Expression Rank

Fig. S1: Generation of input features and labels. (**A**) Composite plot of average 5hmC normalized signal per bin in each expression group across an aggregate gene body and promoter-associated bins. Shadows around the mean is the 95% Confidence Interval (CI). The CI was calculated by multiplying the standard error by the t-value corresponding to 97.5% of the t-distribution, with degrees of freedom of 10847 for Class 1 and 10848 for Class 2. (**B**) Expanded view of the TSS. Vertical lines correspond to division into bins with blue lines representing a fixed size bin of 100 bp. (**C**) Illustration of 5hmC enrichment signal (blue tracks) across three levels of mRNA expression (dark-red tracks) in Activated B cells. *Batf* is highly expressed (Top) and has a strong 5hmC signal distribution; in contrast, *Aox3* is not expressed and has no 5hmC signal (Bottom). *Bcl6* is expressed at low levels and has low 5hmC enrichment (Middle). (**D**) Variance of gene expression for each ranking (highest to lowest TPM on x-axis) across the 49 samples. Y axis represent log10(TPM) values and mean expression per ranking is plotted by a solid blue line and with standard deviation shown as lighter blue shade. Dotted horizontal black line corresponds to 1 TPM (or log10(TPM)=0). Dotted red horizontal line and horizontal shade surrounding it correspond to median TPM thresholds used across different samples.

Fig. S2

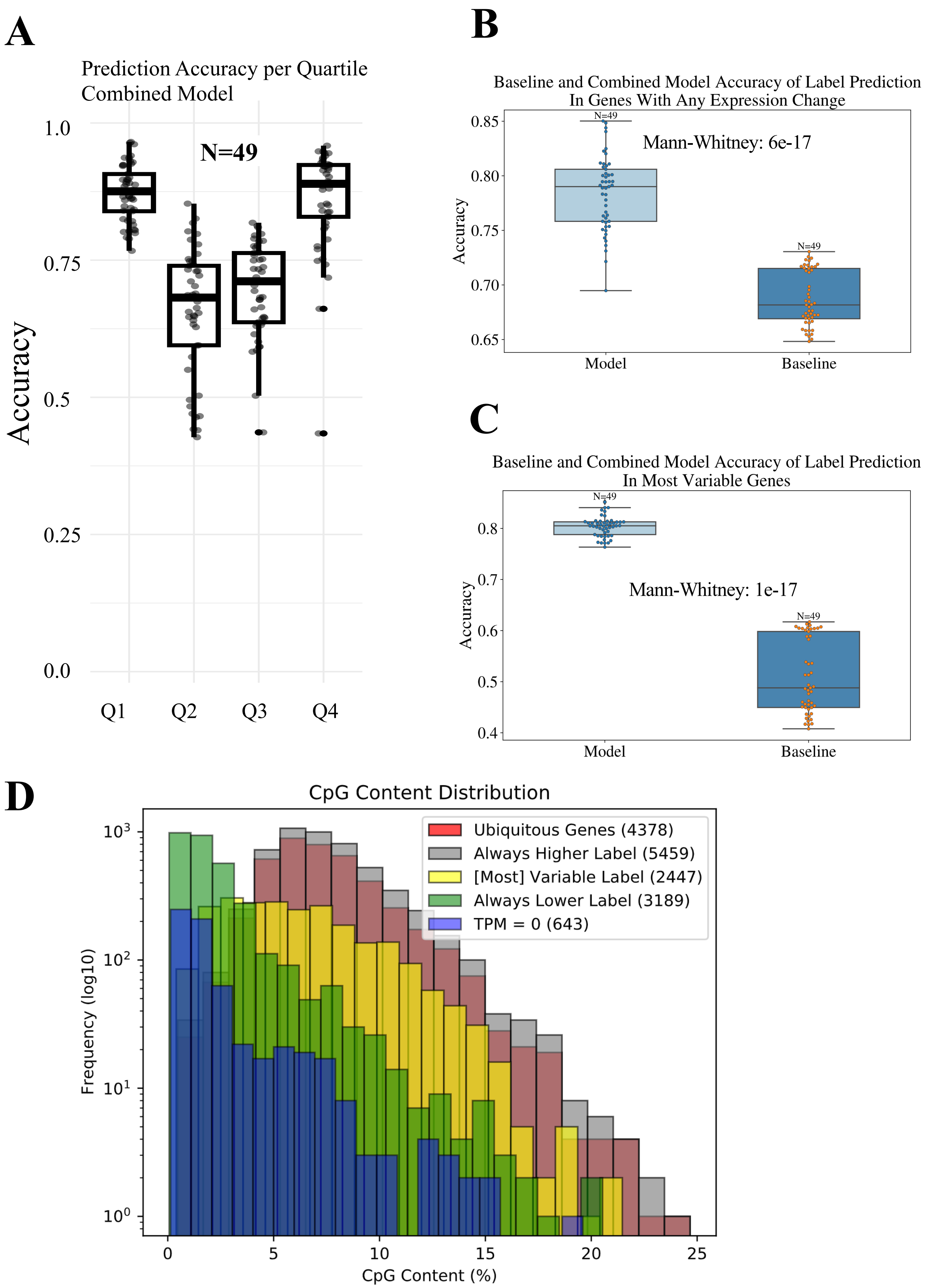
A
Prediction Accuracy per Quartile
Combined Model
N=49
Accuracy
1.0
0.75
0.5
0.25
0.0
Q1
Q2
Q3
Q4
B
Baseline and Combined Model Accuracy of Label Prediction
In Genes With Any Expression Change
Mann-Whitney: 6e-17
N=49
N=49
Accuracy
0.85
0.80
0.75
0.70
0.65
Model
Baseline
C
Baseline and Combined Model Accuracy of Label Prediction
In Most Variable Genes
Mann-Whitney: 1e-17
N=49
N=49
Accuracy
0.8
0.7
0.6
0.5
0.4
Model
Baseline
D
CpG Content Distribution
Ubiquitous Genes (4378)
Always Higher Label (5459)
[Most] Variable Label (2447)
Always Lower Label (3189)
TPM = 0 (643)
Frequency (log10)
10^3
10^2
10^1
10^0
0
5
10
15
20
25
CpG Content (%)

Fig. S2. Assessment of our predictive models and potential confounding factors. (**A**) Prediction accuracy of our combined model across different quartiles of gene expression. Quartiles (Q) were defined using the TPM-normalized gene expression. Q1 represents the bottom 25% of genes based on expression (per sample), whereas Q4 represents the 25% topmost expressed genes. Q1 and Q2 are genes labeled with “Low” expression state, whereas Q3 and Q4 are genes labeled with “High” expression state. (**B-C**) Prediction accuracies of our model versus the majority vote baseline for genes with any variability in their expression state across the 49 samples (**B**) and for genes that are most variable with at least one third of samples having a label opposite to the majority label (**C**). (**D**) CpG content distributions for five different groups of genes classified with respect to their expression levels and states. We calculate CpG content as the number of CpG dinucleotides divided by half the length of the overall sequence.

Fig. S3

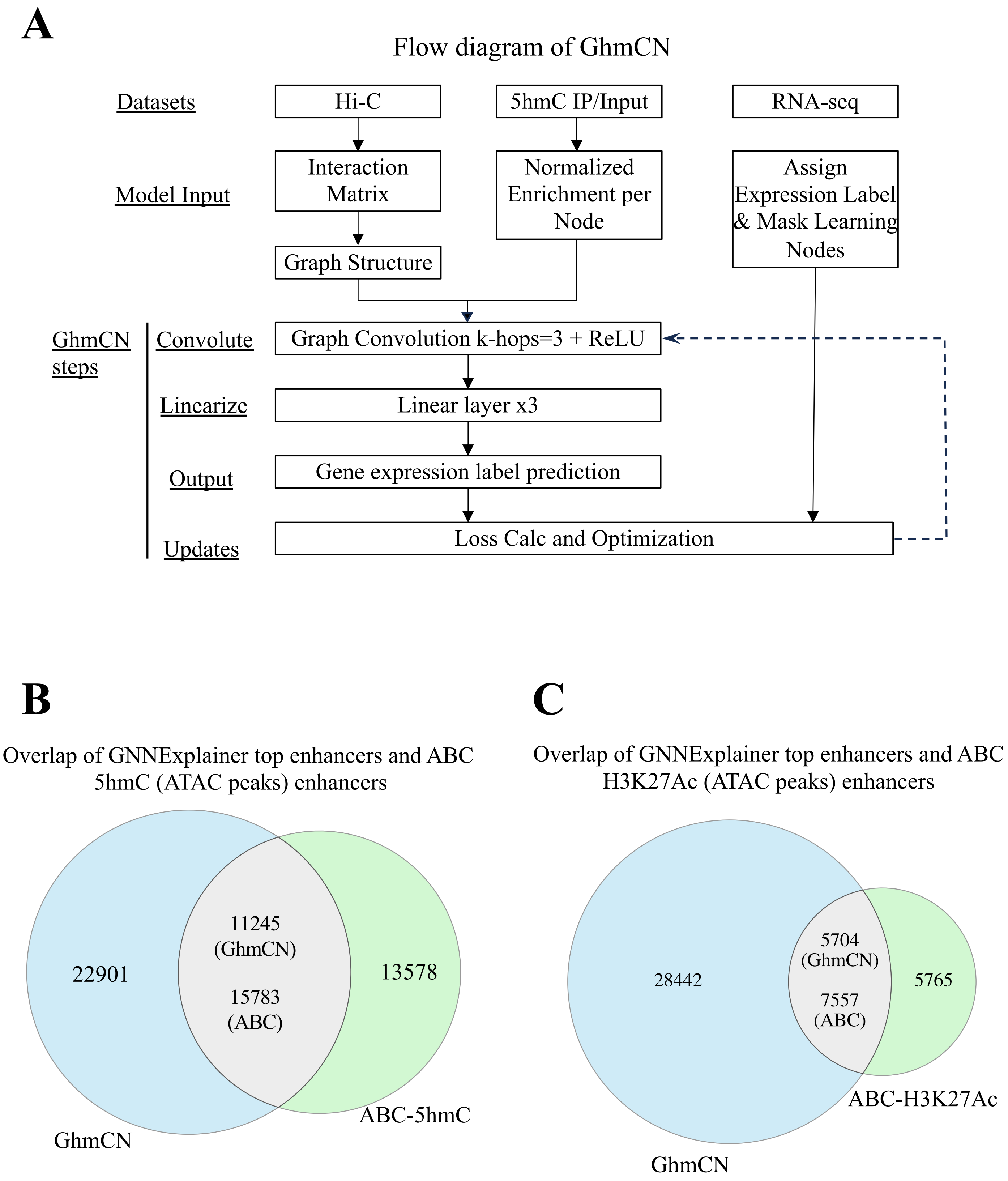
A
Flow diagram of GhmCN
Datasets
Hi-C
5hmC IP/Input
RNA-seq
Model Input
Interaction Matrix
Normalized Enrichment per Node
Assign Expression Label & Mask Learning Nodes
Graph Structure
GhmCN steps
Convolute
Graph Convolution k-hops=3 + ReLU
Linearize
Linear layer x3
Output
Gene expression label prediction
Updates
Loss Calc and Optimization
B
Overlap of GNNExplainer top enhancers and ABC 5hmC (ATAC peaks) enhancers
22901
11245 (GhmCN)
15783 (ABC)
13578
GhmCN
ABC-5hmC
C
Overlap of GNNExplainer top enhancers and ABC H3K27Ac (ATAC peaks) enhancers
28442
5704 (GhmCN)
7557 (ABC)
5765
ABC-H3K27Ac
GhmCN

Fig. S3. Schematic representation of the GhmCN model. (**A**) Flow diagram of the GhmCN method and model architecture. (**B**) Overlap between regions predicted from GhmCN and from ABC-5hmC models for activated B cells. (**C**) Overlap between regions predicted from GhmCN and from ABC-H3K27ac models for activated B cells.

Fig. S4

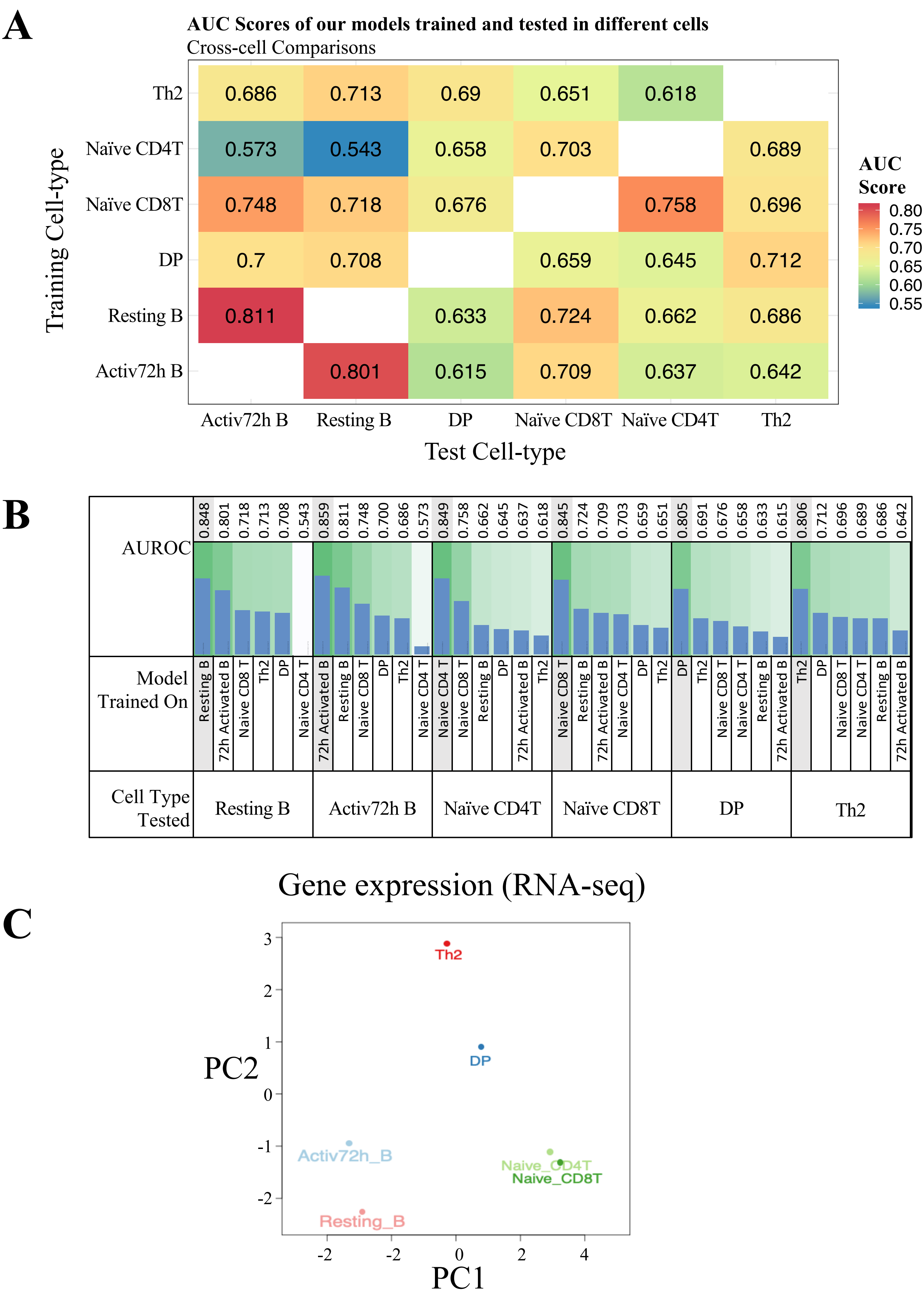
A
AUC Scores of our models trained and tested in different cells
Cross-cell Comparisons
Training Cell-type
Th2
Naïve CD4T
Naïve CD8T
DP
Resting B
Activ72h B
0.686 0.713 0.69 0.651 0.618
0.573 0.543 0.658 0.703 0.689
0.748 0.718 0.676 0.758 0.696
0.7 0.708 0.659 0.645 0.712
0.811 0.633 0.724 0.662 0.686
0.801 0.615 0.709 0.637 0.642
Activ72h B
Resting B
DP
Naïve CD8T
Naïve CD4T
Th2
Test Cell-type
AUC Score
0.80
0.75
0.70
0.65
0.60
0.55
B
AUROC
0.848 0.801 0.718 0.713 0.708 0.543 0.859 0.811 0.748 0.700 0.686 0.573 0.849 0.758 0.662 0.645 0.637 0.618 0.845 0.724 0.709 0.703 0.659 0.651 0.805 0.691 0.676 0.658 0.633 0.615 0.806 0.712 0.696 0.689 0.686 0.642
Model Trained On
Resting B
72h Activated B
Naive CD8 T
Th2
DP
Naive CD4 T
72h Activated B
Resting B
Naive CD8 T
DP
Th2
Naive CD4 T
Naive CD4 T
Naive CD8 T
Resting B
DP
72h Activated B
Th2
Naive CD8 T
Resting B
72h Activated B
Naive CD4 T
DP
Th2
DP
Th2
Naive CD8 T
Naive CD4 T
Resting B
72h Activated B
Th2
DP
Naive CD8 T
Naive CD4 T
Resting B
72h Activated B
Cell Type Tested
Resting B
Activ72h B
Naïve CD4T
Naïve CD8T
DP
Th2
C
Gene expression (RNA-seq)
PC2
3
2
1
0
-1
-2
Th2
DP
Activ72h_B
Naive_CD4T
Naive_CD8T
Resting_B
-2
-2
0
2
4
PC1

Fig. S4. Detailed analysis of the GhmCN model and cross-cell-type predictions. (**A**) AUC values from training GhmCN model on one sample and predicting gene expression in another. (**B**) Same AUC values as presented in (**A**) but organized differently. (**C**) PCA plot of RNA-seq data used in this study. The top 1000 most variable genes among all six samples were used for this analysis.
