# Supplementary material for: Predicting gene expression state and prioritizing putative enhancers using 5hmC signal
Source: Genome Biol. 2024 Jun 3;25:142. doi: 10.1186/s13059-024-03273-z (PMC11145787; doi:10.1186/s13059-024-03273-z)
Supplement: Supplementary file 3 — Additional file 3: Review history. [file 13059_2024_3273_MOESM3_ESM.docx]

Review History

**First round of review**

**Reviewer 1**

**Were you able to assess all statistics in the manuscript, including the appropriateness of statistical tests used?**

Yes, and I have assessed the statistics in my report.

**Comments to author:**

In this study, the authors explored how 5-hydroxymethylcytosine (5hmC), an epigenetic modification of cytosines in DNA, can discriminate between low and high gene expression levels. They found that 5hmC levels can predict these two gene expression categories across 40 different cell types using neural network models. Using cross-cell type testing, the study highlighted a potentially different relationship between 5hmC and gene expression in embryonic stem cells and differentiated immune cells. Furthermore, the authors implemented a graph convolutional neural network to combine 5hmC signals and chromatin conformation data (Hi-C) to predict gene expression categories. By decoding the graphical models, the authors identified new potential regulatory regions exhibiting key enhancer attributes and identified potentially new

enhancer-gene links. The manuscript is overall very well written and addresses a very interesting topic. The applications of the developed models could interest the community and inform new biological discoveries.

Nevertheless, the authors' goal to predict two categories of expression (low and high) is a bit artificial since the thresholds used will vary between cell types and potentially reflect distinct biology. Importantly, we believe a more in-depth assessment of the methodology and comparisons to existing tools would be necessary to further support the claims. Finally, the authors should tone down some of their points as they are not (yet) supported by the data presented in this manuscript.

Major comments:

1. The authors focus on two categories of gene expression: lowly or highly expressed.

a. This dichotomy is very artificial, and it is unclear what biology it reflects since the median threshold could vary between cell types. The authors should provide some distributions of the gene expression values to ensure two categories exist; if not, why would it be relevant to look at these two artificial categories? Recent efforts in the field try to predict gene expression values instead of two categories.

b. The authors alternate between high/low and ON/OFF to refer to the two categories in the text and the figures. We argue that none is correct; maybe lower/higher would better reflect the dichotomy used.

c. Since the authors focus on two categories, it should be reflected in the title and abstract instead of mentioning "gene expression", which can be assumed to be equivalent to gene expression values for us.

2. We would like to see a deeper assessment of the methodology by

a. looking at the variability across different splits of the datasets in training/validation/test. The current results derive from a single split, and it is unclear how much the values presented would vary if different sets of genes would have been selected. All metrics should be computed across several splits to provide some confidence intervals.

b. making sure no information leakage is present. Indeed, with the current strategy, if genes are close enough, some bins might be present in both the training/validation and test sets. Holding out full chromosomes would avoid this issue.

3. The authors claim that their predictive model was comparable to or better than DeepChrome and AttentiveChrome, but it is unclear to us whether the same sets of genes were used for training/validation/test. If it is not the case, the authors should compare their method to these SOTA methods on the exact same datasets to show that their method using 5hmC is possibly better. Even if it is true, it does not inform the readers whether the difference comes from the modelling or the data type used.

4. How does gene expression variability influence the results? Recent studies highlighted that highly expressed genes tend to be less variable between samples and individuals (see https://elifesciences.org/articles/80943 and https://www.embopress.org/doi/full/10.15252/msb.20209539).

5. How do the authors ensure no bias in CpG composition is introduced between the sets? Since 5hmC is to be found at CpG, could CpG composition at promoters, enhancers, and gene body influence the prediction task?

6. The following claim is not supported by the current data and should be toned down: "Our method has several applications: [...], deconvoluting cell composition and tissue of origin in mixed samples where only DNA is available." While these are reasonable hypotheses, the current work does not show that directly.

7. The validation of the prediction of important enhancer-promoter associations is currently very limited, highlighting two cherry-picked examples. A large-scale comparison of the predictions with predictions from ABC (and possibly other methods) is necessary to claim that GhmCN predicted relevant associations overall.

Minor comments:

1. The authors used the top 10 interactors for each window in their graph convolution neural network approach. However, this choice is arbitrary and can miss useful information. We understand there is a computational limitation to the number of interactions the model can incorporate. Nevertheless, we believe it could be relevant to use the strongest interactions overall instead of the top 10 per window (where some might be very weak for a window and other very strong connections for another will be missed).

2. For reproducibility reasons, it would be optimal if the authors specified the versions for all dependencies. While there is a .yml file in their GitHub repository, it does not cover the installation of R, its dependencies and their versions. Addressing this is important to ensure future users can run GhmCN without problems related to software versions.

3. The authors refer to tables we could not find in the submitted manuscript.

4. Some portions of the text are almost identical across sections. See below for two examples:

Example 1:

[Section: Introduction]

"Genomics studies that analyzed long-range interactions and their dynamic association with epigenetic modifications during cell activation and differentiation have in many cases identified novel enhancers. Examples include Enformer (Avsec et al., 2021), which makes gene expression predictions from DNA sequences by integrating information from interactions in the genome that are up to 100 kb away; Activity by Contact (ABC) model (Fulco et al., 2019), which scores enhancer-gene connections to predict enhancers and their target genes by the use of chromatin accessibility or histone acetylation and chromatin conformation; TargetFinder (Whalen et al., 2016), which models the interaction status of predefined pairs of enhancers and promoters by integration of multiple genomic features;

[Section: Integrating distal 5hmC signals in the prediction of gene expression using Graph Convolutional Network (GCN)]

"Analysis of long-range interactions, and their dynamic association with epigenetic modifications during cell activation and differentiation, has been the focus of many genomics studies. In many cases these studies have led to the discovery and validation of novel enhancers. Recent examples include Enformer (Avsec et al., 2021), which makes gene expression predictions from DNA sequences by integrating information from interactions in the genome that are up to 100 kb away; Activity by Contact model (Fulco et al., 2019), which constructs enhancer-gene connection maps to predict enhancers and their target genes by the use of chromatin accessibility or acetylation and chromatin conformation; TargetFinder (Whalen et al., 2016), which models the interaction status of predefined pairs of enhancers and promoters by integration of multiple genomic features; among others."

Example 2:

[Section: Introduction]

This structure makes use of the GraphSAGE framework (Hamilton et al., 2017), which allowed us to train an embedding-generator function on one cell-type, and then to use this function in a previously unseen cell type. We argued that as long as the graphs for each of our samples, as well as the node attributes (such as 5hmC enrichment), were generated the same way, the trained function would retain predictive value across different cell types."

[Section: Integrating distal 5hmC signals in the prediction of gene expression using Graph Convolutional Network (GCN)]

"This GCN approach makes use of the GraphSAGE framework (Hamilton et al., 2017), which allows us to train an embedding-generator function in a cell-type, and then use this function in a previously unseen cell-type. We anticipated that, as long as the graphs and the node attributes (such as 5hmC enrichment and Input signal), are generated similarly for each sample, the trained function may retain predictive value across different cell types."

5. We detected some typos in the text (e.g. Materials and Methods - Analysis of 5hmC Enrichment datasets. [...] For each gene longer than 1000bpwe; Page 6, line 13, "can be used to effectively to predict gene expression…")

6. Figure legends should be revised as some references point to the incorrect figure. For example, Supplementary Figure 1A specifies the presence of a dashed diagonal green line, which cannot be found in the figure.

7. Page 2, lines 1-2, the authors claim that "DNA methylation is a generally heritable mark that is quickly restored at most sites…". We did not find support for this claim in the referenced study. On the contrary, other studies showed that DNA methylation and demethylation are slow processes, and the turnover for these processes takes days-weeks (see e.g. https://doi.org/10.1038/s41467-020-16354-x).

8. We would suggest that the authors modify the titles of the subsections in the Results section to provide the main message of each subsection instead of generic/methodological titles.

9. Page 5, lines 15-16, "samples not yet seen by the model (...Fig. 1D; Table S8, see "genes withheld" row results compared to "cross-cell type" row results". There is no such labelling as 'Cross-cell type' in Table S8.

10. There is a mixed-use of '5hmc' and the standard '5hmC'.

11. Page 6, lines 21-31, is a repetition of the introduction. Moreover, we believe the message is better in the Discussion section.

12. Page 7, lines 6-8, the support to your claim is not clear from the text, perhaps you should explicitly refer to 'Fig 3D, blue columns'

13. Page 8, lines 3-11, should be merged with the previous section

**Reviewer 2**

**Were you able to assess all statistics in the manuscript, including the appropriateness of statistical tests used?**

Yes, and I have assessed the statistics in my report.

**Comments to author:**

This work studies the effects of 5-hydroxymethylcytosine (5hmC) on gene expression. The authors first use simple machine learning models, such as logistic regression, SVM, random forests (RF), and fully connected deep neural networks (FCDNNs) to predict binary gene expression values (ON/OFF) based on 5hmC signals in gene bodies, their vicinities, and promoters. They show that these models have high AUROCs, and their feature attribution based on DeepLIFT reveal important bins for gene expression. It turns out that the most important bins are around the TSSs of the genes, especially in downstream of the TSSs. It is shown that the models can be effectively generalized to other unseen cell types. Finally, in order to identify functional enhancers of genes based on 5hmC signals, the authors use ICE-normalized Hi-C data, extract the graphs based on top-10 interactions, and employ graph convolutional networks (GCNs) to predict the binary gene expression values from the 5hmC signals in the genomic bins of 10Kb, the resolution of the Hi-C data. They then use GNNExplainer to find the important nodes and edges of the graphs, among which there are important and validated enhancers (TetE1 and TetE2) of Aicda in activated B cells. To answer if the GCN models can be used in the cells without Hi-C data, they generate average Hi-C and train the models based on that and show that although there are some performance reductions, the prediction accuracies are reasonable. Overall, this work studies how 5hmC signals in gene proximal and distal enhancer regions can affect gene expression in different cell types. It can also be used to nominate functional enhancers of genes when only 5hmC and Hi-C data are available.

Comments:

- Why are genes classified as ON/OFF? I think working with signal values in a regression model could be more informative than binarizing the signals.

- Are differences between AUC distributions in Fig. 1 significant?

- Why DNNs are trained up to the epoch 60? I wonder if any early stopping criteria based on validation set have been used to prevent overfitting? How about GCN models?

- What is 5hmC-enrichment profile per bin in GCN models? Is it a scalar or vector? Fig. 3A shows it as a vector. If so, in what resolution these signals are generated, 100bp?

- Can you also plot Precision-Recall curves in Figs. 3B-C?

- Are the reductions of AUCs from cell-type-specific to averaged Hi-C seen in Figs. 3B and 3C significant?

- Again, are the AUPRC and AUROC differences when using 10-nearest bins, as shown in Fig. 3D significant? It would be better to plot the distributions (for example, box plots) instead of a scalar score. The authors should perform rigorous statistical tests in all comparisons of the paper to show the significance scores.

- ICE normalization can suffer from distance effect. Have you tried other methods that account for distance effect (like HiC-DC+)? The same issue exists in Fig. 5A. It seems most of the top-10 interactions are the nearby bins. I would suggest creating graphs based on significant interactions of HiC-DC+ and compare the results with the graphs extracted based on top-10 interactions.

- It is not clear how the graphs are built from the Hi-C data. Are these graphs per chromosome? How many nodes and edges are there in each graph? What are the degree distributions of gene and non-gene nodes? This information can help better understanding of the GCN models.

- The authors refer to GC-MERGE (Bigness 2022) for the GCN models used. It would be helpful to provide a simple schematic of model architecture, inputs, and outputs.

- There is not any comparison between DNN models (which only uses 5hmC signal in the gene bodies and their vicinities) and GCN models (which uses 5hmC signals in all genomic bins connected to the genes based on Hi-C interactions). This is a very interesting benchmark to see how much Hi-C and 5hmC signals in enhancers can add to the predictive power of the models.

- Regarding "Since 5hmC is a stable, covalent DNA modification, assessing 5hmC signals would enable the study of such samples and can also provide estimates on differences of gene expression and enhancer utilization across different conditions", is Hi-C data available in these samples? If not, the proposed models cannot be used to identify the enhancers.

Minor comments:

- Page 14, line 32: the yellow highlight cannot be seen in Fig. 5. Was it supposed to be pink?

- Page 14, line 38: H3K27ac is repeated.

- Page 16, line 54: table S8 should probably be table S9.

**Reviewer 3**

**Were you able to assess all statistics in the manuscript, including the appropriateness of statistical tests used?**

Yes, and I have assessed the statistics in my report.

**Comments to author:**

Gonzalez-Avalos present two neural network-based approaches (FCDNN and GCN) for predicting discretized gene expression from 5hmC signals in different genomic regions (gene body, TSS, enhancers for GCN). As stated by the authors in the abstract and Discussion, this model performs comparable to existing models using genomic data and histone marks for the same purpose. The authors analyze in detail the across cell type performance of their approach and demonstrate the interpretability of their model to find important 5hmC signals and putative novel enhancers based on GCNs. As the 5hmC signal has been neglected in many previous approaches, I consider this study highly interesting for the community. However, I have several important concerns regarding the presentation of the method and reproducibility and the comparison to previous approaches as detailed below.

Major comments:

1. Section "Within sample predictive models of gene expression from 5hmC enrichment" does not give detailed enough information about the model inputs, which are neither clarified in Methods. Specifically, what are the inputs of the different machine learning methods? In Fig. 1A, there are "bins" (?) 1 to 230. What do those represent? Are these genomic bins? If yes, which regions do they refer to?

2. Section "Cross-cell type comparisons with GhmCN" (and also Methods) is lacking information, how exactly information is transferred from one one cell type to another. As I understood from the current version of the manuscript, the "input features" also include the graph structure (which includes Hi-C contacts as nodes). If the Hi-C contacts in another cell type are different, how can the network (parameters) be transferred to that cell type?

3. For case study A, I wondered what we gain from the (rather complex) GhmCN model compared to a simple approach, where we first ranked the Hi-C contacts (as done for the network anyway) and then testing contacting regions of their 5hmC signal. Would such a simple approach be equally suited to propose putative enhancer regions?

4. The authors state in the abstract that they "show that 5hmC levels alone can predict gene expression with a predictive power comparable to models that utilize combinations of histone modifications" and in Discussion "Also, our predictive power was comparable or better with respect to existing models that use the genomic distribution of multiple histone marks [...]"

However, I did not find a direct comparison to such methods in the current version of the manuscripts. Specifically, the authors report AUC values from previous studies in the Discussion. However, I do not consider these comparable to the AUC values reported in this manuscripts. As the authors show, performance varies greatly even between the cell types studied in this manuscript. In addition, there are additional variables (e.g., discretization thresholds) that might impair a direct comparison. Hence, I consider a direct comparison necessary to make such a statement. Otherwise, the statement (which is in some sense central to this manuscript) is not supported by the data shown.

5. The discretization approach (as far as I understand) the authors chose (based in the median gene expression) implicitly assumes that genes are "On" in half of the samples/conditions, which will likely be false for cell type-specific genes on the one end of the scale and "house keeping" genes on the other end. If I misunderstood the labeling and this is based on the global median (across all genes and samples), my minor comment regarding RPKM/TPM becomes more relevant.

Minor comments:

6. From my perspective, the first section of Results (Compilation of 5hmC and gene expression datasets) does not present actual results but rather data processing and should be transferred to the Methods section.

7. In some of the sub-figures of Fig. 1, legends are unnecessary, because the same labels are already provided at the x-axis.

8. In section "Aggregate predictive models of gene expression from 5hmC enrichment" (line 61), the authors state that the use "training and test splits similar to the Whole model." Why did they not use exactly identical splits? If they did, numbers (AUC, F1) would be directly comparable.

9. In Fig. 1C, the authors draw AUC values from ESC cells as red dots over the boxplot for the "Whole" model. For a direct comparison the the "ESC" model, an additional boxplot or, even clearer, a scatter plot of "Whole" vs "ESC" AUC values would be more informative.

10. In the sub-title of Fig. 1D, the other use the term "cross-validations" (which is typically understood differently in the field), whereas in the text they use "cross-cell type". I would suggest to also use this term in the sub-title of Fig. 1D for clarity.

11. The authors evaluate their GhmCN models in the cross cell type setting and find that AUC values are substantially worse for some combinations of training and test cell type. I wondered if Hi-C information has been even harmful for model performance in such cases (relative to a model that does not use Hi-C information).

12. In Fig. S3B/C, we see that the ranking of model performance (AUC) across cell types often coincides with the similarity of their expression profiles. However, this does not seem to be the case for naive CD8T (test) and naive CD4T (train), whereas vice versa the ranking is as expected. Could the authors comment on possible reasons for this observation?

13. In section "Explaining the predictions of our GhmCN models," the authors state that "the top ranked nodes for each gene showed a high probability of containing regulatory elements with biological significance." However, I did not find any Figure associated with that section that supports this statements. Currently, I assume that this is rather related to the structure of the manuscript, and this is shown in the subsequent case studies. Please clarify.

14. In Fig. 4A (and partly also in Fig. 5) some labels and also the scale (genomic positions) are hard to read and in some cases partly cropped.

15. In case study B, the authors state that "Both ReMap 2022 and UniBind provided further evidence for BATF and IRF4 binding as well as general bZIP TF binding in Kif3a-A and Kif3a-B.", which I could not spot from Fig. 5. Please clarify where that information is represented.

16. In Material and Methods, "Analysis of RNA-seq datasets", the authors describe that they used RPKM-normalized expression values. Although it might not make a (major) difference for discretizing based on the median, I wondered why the authors decided against (more established, from my perspective) TPM values.

17. In Material and Methods, "Analysis of RNA-seq datasets", they authors term the gene labels as "High" and "Low", whereas in Figures and other parts of the manuscript, these are called "On" and "Off". While I consider the first term more appropriate, I would at least recommend to use consistent labels.

18. The implementation of Deep Neural Networks (FCDNN) seems to be missing from the github repository.

19. While the authors explicitly state (Materials and Methods, Deep Neural Networks) that FCDNNs are implemented using TensorFlow (NB: which does not belong, but is only implemented in Python) and Keras, this information (pytorch according to the github repo) is missing for the GCNs.

20. Additional details should be given for the application of GNNExplainer to the GCNs. Currently, this is only one sentence in section "Graph Convolution Networks."

21. Typos: Materials and Methods, "Analysis of 5hmC Enrichment datasets," line 28: 1000bpwe -> 1000bp we

**Authors Response**

**Point-by-point responses to the reviewers’ comments:**

# Reviewer #1:

***NOTE:*** *We have colored newly added or modified text with blue font throughout the main text. For this response document, the reviewer comments are in red font, our responses are in black font and quotes and copy/pastes from the manuscript are in blue font.*

1. The authors focus on two categories of gene expression: lowly or highly expressed.

a. This dichotomy is very artificial, and it is unclear what biology it reflects since the median threshold could vary between cell types. The authors should provide some distributions of the gene expression values to ensure two categories exist; if not, why would it be relevant to look at these two artificial categories? Recent efforts in the field try to predict gene expression values instead of two categories.

*Reply: We understand the concern raised by this reviewer. We now changed our title (and all the text) to reflect that we are predicting “expression state” rather than expression. We also now provide a figure that plots the distribution of mean gene expression and gene expression variance showing that median is a good estimate of switch from high gene expression variance to low (Supp Fig 1D - below). We have tried other splits such as grouping all zeros plus the lowest 25th percentile as low and the rest of the genes 25-100th percentile as high (Unbalanced) and seen that predictive performance of these categories have fallen compared to the balanced data split using median (Reviewer Fig 1.1). Therefore, we believe our way of doing this is justifiable to an extent and is in line with previous work in the field (e.g., DeepChrome).*

*We agree that predicting gene expression values is a more relevant problem, however, doing this with a single track of epigenomic information is unlikely to be competitive with the recent approaches integrating hundreds of such tracks to perform the same task. We hope this will inspire future work by us and others for the integrated utility of 5hmC with other genomic tracks and DNA sequence to predict gene expression values or other functional genomic elements.*

**Supp Figure 1D:** Variance of gene expression for each ranking (highest to lowest TPM on x-axis) across the 49 samples. Y axis represent log10(TPM) values and mean expression per ranking is plotted by a solid blue line and with standard deviation shown as lighter blue shade. Dotted horizontal black line corresponds to 1 TPM (or log10(TPM)=0). Dotted red horizontal line and horizontal shade surrounding it correspond to median TPM thresholds used across different samples.

**Reviewer Figure 1.1:** Comparison of AUC distributions across 49 samples for sample-specific models that either use the median TPM (balanced) or categorizes all zero expression genes plus the lowest expressing 25% of the remaining genes as Low and the 25 to 100% percentile as High.

b. The authors alternate between high/low and ON/OFF to refer to the two categories in the text and the figures. We argue that none is correct; maybe lower/higher would better reflect the dichotomy used.

*Reply: Thank you for bringing up this discordance. We have now corrected this to “High/Low” expression throughout the text.*

c. Since the authors focus on two categories, it should be reflected in the title and abstract instead of mentioning "gene expression", which can be assumed to be equivalent to gene expression values for us.

*Reply: We understand that our utilization of the term can cause confusion, so we now updated both the title and the abstract (as well as revised the rest of the manuscript) to state that we are predicting “gene expression state”.*

2. We would like to see a deeper assessment of the methodology by

a. looking at the variability across different splits of the datasets in training/validation/test. The current results derive from a single split, and it is unclear how much the values presented would vary if different sets of genes would have been selected. All metrics should be computed across several splits to provide some confidence intervals.

*Reply: Thank you for the suggestion to assess the methodology's robustness by examining the variability across different splits of the datasets in training/validation/test. We have now performed this analysis by leaving out two autosomes at a time (chrs 1 to 19) and developing 19 different Combined models, each utilizing all 49 samples. We then plot, for each held out test chromosome, the distribution of AUC values across these 49 samples giving us a distribution of test AUCs (boxplot) for each chromosome. As the below plot shows (Fig. 1D), across these different splits, the model performance is quite robust across all chromosomes. In terms of different samples, we see one outlier sample that is Th2 cells, which also was the outlier in our other assessments. We did not want to arbitrarily remove Th2 cells from our analysis and decided to report the results as is.*

**Figure 1D:** AUC score distributions to assess the robustness of the combined model approach by leaving out a different chromosome for testing each time. We trained 19 different models each with a different set of excluded test and validation chromosomes, indicated in the X-axis. Each box plot shows the distribution of the AUC scores calculated for the test chromosome across 49 different samples. The combined model with the ROC curve reported in panel C is highlighted with a red box and its overall AUC is depicted by the horizontal dashed line.

b. making sure no information leakage is present. Indeed, with the current strategy, if genes are close enough, some bins might be present in both the training/validation and test sets. Holding out full chromosomes would avoid this issue.

*Reply: We appreciate the consideration of data leakage in our analysis. We are happy to clarify that, in our work, we took precautions to avoid information leakage by holding out entire chromosomes from predictions of gene expression from 5hmC data. This approach aligns with best practices, and we referenced the observations in the paper 'Pitfalls of Machine Learning,' which emphasized the importance of separating whole chromosomes to mitigate data leakage.*

*To ensure this, we used the genes located in chr4 for testing purposes and the genes in chr5 for validation/development/parameter hyper tuning. This choice was made considering that both chromosomes are of “medium” size and have a similar number of total protein-coding genes (1316 and 1340 genes, respectively). As we have now presented above and in* ***Fig 1D*** *in the main text, we repeated this by leaving out one test (each of the 19 autosomes) and one validation (randomly chosen) chromosome to obtain 19 different models showing the performance is robust to different splits.*

*In addition, another type of data leakage could have been the use of multiple replicates of 5hmC data for each sample type (e.g., mESC with over 10 replicates) with some replicates used in training and others for testing. After our new QC and filtering, we also eliminated the use of multiple replicates by keeping one high-quality replicate for each sample type leaving us with 49 paired 5hmC-RNAseq data to work with. Overall, we believe, we have taken all the cautionary steps to avoid multiple different ways of possible data leakage. We thank the reviewer for their suggestions about this.*

3. The authors claim that their predictive model was comparable to or better than DeepChrome and AttentiveChrome, but it is unclear to us whether the same sets of genes were used for training/validation/test. If it is not the case, the authors should compare their method to these SOTA methods on the exact same datasets to show that their method using 5hmC is possibly better. Even if it is true, it does not inform the readers whether the difference comes from the modeling, or the data type used.

*Reply: We appreciate the reviewer’s insightful observation regarding the need for a more in-depth comparison to state-of-the-art (SOTA) methodologies, specifically DeepChrome and AttentiveChrome. The reviewer rightly points out the importance of ensuring comparability in terms of the sets of genes used for training/validation/test. It’s a valid concern whether any observed differences stem from the modeling approach or the data type employed.*

*In response, we would like to clarify that the datasets used by DeepChrome and AttentiveChrome are a number of commonly used human cell lines some of which do not have 5hmC profiles. Fully differentiated cell types and cell lines have very low levels of 5hmC, which makes them not an ideal model for studying the role of 5hmC in gene regulation. Therefore, we chose to remove direct comparisons to DeepChrome and AttentiveChrome in this revision.*

*Nevertheless, we recognize the importance of putting our work in the context more comprehensively. We now included a whole section in the manuscript entitled “Further assessment of our predictive models and potential confounding factors” which also includes a new baseline model that uses a majority voting strategy and gets to memorize (unlike our method) gene expression across training samples. In addition, for the second part, we performed a comparison with the ABC method, and we expanded our work in a new section to provide additional insights into our findings. We believe that this adjustment enhances the clarity and completeness of our comparative analysis.*

4. How does gene expression variability influence the results? Recent studies highlighted that highly expressed genes tend to be less variable between samples and individuals

(see [https://elifesciences.org/articles/80943](https://urldefense.com/v3/__https:/elifesciences.org/articles/80943__;!!LF7Dpc92lww!7f3pmyJbRTbWI7W_lGV0vT5CtcvXw76niJ_KduErto1X4N9QAGa1c67jOhN2o-6t_IiUgB_rRFMN5I02$) and [https://www.embopress.org/doi/full/10.15252/m sb.20209539)](https://urldefense.com/v3/__https:/www.embopress.org/doi/full/10.15252/msb.20209539__;!!LF7Dpc92lww!7f3pmyJbRTbWI7W_lGV0vT5CtcvXw76niJ_KduErto1X4N9QAGa1c67jOhN2o-6t_IiUgB_rRPNDw8rR$).)

*Reply: We appreciate the reviewer's insightful consideration regarding the potential influence of gene expression variability on our results. In response to this concern, we conducted a comprehensive analysis by plotting the Mean versus variance of the sorted TPM expression values as suggested. Our findings indicate a positive correlation, wherein higher TPM values are associated with lower expression variance across samples (see Supp Figure 1D above and Reviewer Figure 1.2 below). Our analysis with respect to accuracy of predictions across different expression quartiles highlighted that the High/Low expression state for the middle quartiles 25-50% and 50-75% are more challenging to predict as expected. This was mainly due to these quartiles harboring genes with TPMs closer to the median threshold rather than their expression variability.*

**Reviewer Figure 1.2:** Plotting gene expression variability against mean expression for our 49 samples as was done by Einarsson et al Figure 1E.

**Supp. Fig 2A:** Prediction accuracy of our combined model across different quartiles of gene expression. Quartiles were defined using the TPM-normalized gene expression. Q1 represents the bottom 25% of genes based on expression (per sample) mostly composed of non-expressed genes, whereas Q4 represents the 25% topmost expressed genes.

5. How do the authors ensure no bias in CpG composition is introduced between the sets? Since 5hmC is to be found at CpG, could CpG composition at promoters, enhancers, and gene body influence the prediction task?

*Reply: We appreciate the reviewer bringing up an important point that we now analyzed in much greater detail. First of all, since we do not directly use the sequence information and that we make sure to hold out entire chromosomes to avoid memorization and data leakage, our models will not have a way to explicitly utilize such information.*

*However, we now performed multiple different lines of investigations that hopefully will clarify the potential interplay among 5hmC, CpG content and gene expression:*

*We then compared the CpG content distributions of promoter regions (+/- 1kb around the TSS) for these groups and observed substantial differences (Supplementary Fig. 2D)). As previously documented (Cheng et al., 2012), we observed that CpG content of the promoter has a positive correlation with gene expression (e.g., highest overall CpG content for genes labeled Always High). However, since we avoid memorization of constitutive features in our DNN model by leaving out entire chromosomes from the training, this sequence content bias does not become an obvious pitfall for our approach. Our above-mentioned* *performance for genes with variable expression states across samples also suggests our model’s ability to incorporate the cell-typespecific modification information as intended.*

**Supp. Fig 2D:** CpG content distributions for five different groups of genes classified with respect to their expression levels and states. We calculate CpG content as the number of CpG dinucleotides divided by half the length of the overall sequence.

*Given the above findings concerning the importance and contribution of cell-specific and sequence-based features of the promoter regions, we performed one last evaluation by removing any bin surrounding*

*the promoter region (130 total bins surrounding TSS) from the 5hmC feature set. Although we observed a drop in predictive performance when bins surrounding the TSS are hidden from the model training (accuracy from 0.79 to 0.73 and AUC from 0.87 to 0.83), there remains substantial predictive power in 5hmC features of bins representing the gene body independent of the promoter region. See below for pictorial representation of these results (Reviewer Figure 1.3).*

**Reviewer	Figure	1.3:**

Prediction performance with and without 5hmC features of bins proximal to the promoter/TSS region.

6. The following claim is not supported by the current data and should be toned down: "Our method has several applications: [...], deconvoluting cell composition and tissue of origin in mixed samples where only DNA is available." While these are reasonable hypotheses, the current work does not show that directly.

*Reply: We appreciate the reviewer's comment and agree that, while there are many potential exciting applications, our current work does not demonstrate their feasibility. Therefore, we amended our abstract as follows:*

*“Our work highlights the importance of 5hmC in gene regulation through proximal and distal mechanisms and provides a framework to link this DNA base modification to genome function. With the recent advances in 6-letter DNA sequencing by short and long-read techniques, profiling of 5mC and 5hmC may be done* routinely across many samples in the near feature, hence, providing a broad range of applications for the methods we propose here and their extensions.”

*Note that we kept our Discussion section about these potential applications but there we clearly indicate that these are future directions by starting that paragraph with “As a future direction …”. We also added a whole new paragraph about the technical and conceptual limitations of our current work in the Discussion section.*

7. The validation of the prediction of important enhancer-promoter associations is currently very limited, highlighting two cherry-picked examples. A large-scale comparison of the predictions with predictions from ABC (and possibly other methods) is necessary to claim that GhmCN predicted relevant associations overall.

*Reply: We appreciate the reviewer's insightful suggestion regarding the validation of enhancer-promoter associations and the need for a more extensive comparison with other methods, such as ABC. In response to this valuable feedback, we conducted a large-scale comparison with the ABC method, using activated B cells which, in addition to 5hmC and H3K27ac, has ATAC-seq and high-depth Hi-C data available. We added a full new section on this entitled “5mC-based Activityby-Contact model identifies novel distal enhancers and their target genes”* *and also a new main figure (Figure 3) - which we are not copy pasting for the sake of space. We are copy pasting, however, the discussion of this new section from the Introduction to provide a summary of our findings from this comparison:*

*“Considering the observed 5hmC enrichment in cell-specific distal enhancers, we were interested in integrating 5hmC with 3D chromatin structure data to prioritize putatively functional enhancer regions for each gene while performing the task of predicting that gene’s expression state. For this, we started with adapting the recently developed Activity-by-Contact (ABC) model (Fulco et al., 2019) to utilize 5hmC signal (ABC-5hmC) instead of H3K27ac (ABC-H3K27ac). For activated B cells, ABC-5hmC captured >89% of the regions identified as putative enhancers by ABCH3K27ac but also reported over 17,000 additional regions with strong 5hmC signal and weaker ATAC-seq peaks. One of the putative elements uniquely captured by ABC-5hmC corresponded to a region that shared 5hmC dynamics with two other validated TET-dependent enhancers of the Aicda gene, the primary regulator of class switch recombination (CSR). On the other hand, ABC-H3K27ac-specific regions were enriched for H3K4me3 signals and TSS proximity."*

*We also carried out an overlap analysis between GhmCN predictions and ABC-5hmC model we developed, which suggested a substantial fraction of predictions were shared but around two thirds of GhmCN predictions at 10 kb resolution did not overlap ABC-5hmC predictions that happen at peak resolution and only are possible for regions harboring ATAC-seq peaks. A comprehensive comparative analysis of such GhmCN bins with ABC predictions, as we have done for ABC-5hmC versus ABC-H3K27ac, is challenging though due to their coarse resolution (10 kb bins).*

**Supp. Fig 3B:** Overlap between regions predicted from GhmCN and from ABC-5hmC models for activated B cells.

**Minor comments:**

1. The authors used the top 10 interactors for each window in their graph convolution neural network approach. However, this choice is arbitrary and can miss useful information. We understand there is a computational limitation to the number of interactions the model can incorporate. Nevertheless, we believe it could be relevant to use the strongest interactions overall instead of the top 10 per window (where some might be very weak for a window and other very strong connections for another will be missed).

*Reply: We thank the reviewer for their suggestion. We indeed repeated our analysis now using most significantly interaction top-10 bins using FitHiC as a tool for significance assignment. This gave us very similar results between GhmCN and FitHiC. The prediction accuracies were nearly identical (Accuracy, AUC, AUPR all within 1% of each other) and when we focused on one of our specific case studies using GNNExplainer on the FitHiC-based Hi-C graph, we got the same 10 regions as prioritized regions for Aicda gene compared to our original GhmCN model with only slight differences in contribution scores.*

**Reviewer Figure 1.4:** Overlap between regions predicted from GhmCN and from ABC-5hmC models for activated B cells.

2. For reproducibility reasons, it would be optimal if the authors specified the versions for all dependencies. While there is a .yml file in their GitHub repository, it does not cover the installation of R, its dependencies and their versions. Addressing this is important to ensure future users can run GhmCN without problems related to software versions.

*Reply: We have added to our GhmCN repository a yml file for the GCN, a yml file for the FCDNN, and a folder with yml files for other analysis, such as running the ABC model, performing the DeepLift analysis,* *performing analysis in R, and more. Moreover, we have deposited into this same repository a folder with the source files to reproduce the FCDNNs.*

3. The authors refer to tables we could not find in the submitted manuscript.

*Reply: We apologize for this and would like to mention that this issue was fixed after the submission but likely not before this reviewer(s) wrote their critique. We now made sure all the tables are part of the submission. We have 5 main and 11 supplementary tables now, for your reference. Thank you for noticing this.*

4. Some portions of the text are almost identical across sections. See below for two examples:

Example 1:

[Section: Introduction]

"Genomics studies ..;

[Section: Integrating distal 5hmC signals in the prediction of gene expression using Graph

Convolutional Network (GCN)]

"Analysis of long-range interactions, ..."

*Reply: Thanks for highlighting this. In this case, we simply removed the whole paragraph from the GCN section.*

Example 2:

[Section: Introduction]

This structure makes use of the GraphSAGE framework …."

[Section: Integrating distal 5hmC signals in the prediction of gene expression using Graph Convolutional Network (GCN)]

"This GCN approach makes use of the GraphSAGE framework (Hamilton et al., 2017..."

*Reply: Thanks for highlighting this. In this case, we believe some of the information was relevant to both sections but we shortened the discussion in the intro and made sure sentences are not repetitive. In addition, we have gone through the overall Introduction one more time and streamlined it and shortened it significantly.*

5. We detected some typos in the text (e.g. Materials and Methods - Analysis of 5hmC Enrichment datasets. [...] For each gene longer than 1000 bp we; Page 6, line 13, "can be used to effectively to predict gene expression…")

*Reply: We appreciate your attention. We revised these typos and thoroughly checked our manuscript for other such cases.*

6. Figure legends should be revised as some references point to the incorrect figure. For example, Supplementary Figure 1A specifies the presence of a dashed diagonal green line, which cannot be found in the figure.

*Reply: We apologize for this, although the green lines were dashed when we plotted them, we agree they do not show up that way in the PDF. We fixed the caption and we double checked to make sure all figure references are correct in this version.*

7. Page 2, lines 1-2, the authors claim that "DNA methylation is a generally heritable mark that is quickly restored at most sites…". We did not find support for this claim in the referenced study. On the contrary, other studies showed that DNA methylation and demethylation are slow processes,	and	the	turnover	for	these	processes	takes	days-weeks	(see

e.g. [https://doi.org/10.1038/s41467-020-16354-x)](https://urldefense.com/v3/__https:/doi.org/10.1038/s41467-020-16354-x__;!!LF7Dpc92lww!7f3pmyJbRTbWI7W_lGV0vT5CtcvXw76niJ_KduErto1X4N9QAGa1c67jOhN2o-6t_IiUgB_rRExI3LGE$).

*Reply: We appreciate the note on this and the reference to Dirk Schübeler’s recent work. As this work also beautifully shows, the kinetics of methylation and its maintenance is far from being uniform across the genome. We now removed the word “quickly” from our sentence and as part of the process, we decided to directly delve into talking about 5hmC rather than talking about 5mC restoration dynamics. Below is how that sentence reads now:*

*“Enhancers that are newly activated during cellular activation or differentiation show progressive deposition of 5hmC and loss of 5mC during activation and differentiation (Lio et al., 2019). 5hmC is a highly stable modification in differentiated non-proliferating cells (Bachman et al., 2014). 5hmC is also strongly enriched in accessible genomic regions (Lio et al., 2019; Lio et al., 2016), as well as in euchromatin and transcribed regions (Lopez-Moyado et al., 2019; Nestor et al., 2012).”*

8. We would suggest that the authors modify the titles of the subsections in the Results section to provide the main message of each subsection instead of generic/methodological titles.

*Reply: We have now modified each result section title to reflect the main conclusions of that section, we thank the reviewer for pointing this out.*

9. Page 5, lines 15-16, "samples not yet seen by the model (...Fig. 1D; Table S8, see "genes withheld" row results compared to "cross-cell type" row results". There is no such labelling as 'Cross-cell type' in Table S8.

*Reply: We have now modified the text and figures to provide a schematic representation of the training/validation/testing split for all different types of models we have trained. This and rewording of the text, hopefully, clarifies this and any similar confusions.*

10. There is a mixed-use of '5hmc' and the standard '5hmC'.

*Reply: We only found one instance of 5hmc in the text and corrected it. Thank you.*

11. Page 6, lines 21-31, is a repetition of the introduction. Moreover, we believe the message is better in the Discussion section.

*Reply: Thanks for highlighting this. In this case, we simply removed this whole paragraph from the GCN section.*

12. Page 7, lines 6-8, the support to your claim is not clear from the text, perhaps you should explicitly refer to 'Fig 3D, blue columns'.

*Reply: We have now reorganized this section and the corresponding figure (now Figure 4) to address this and other concerns about evaluating the statistical significance of reported differences. The subpanel that now supports the claim mentioned above is Figure 4E (pasted below).*

**Supp. Fig 4E:** AUC and AUPR scores to assess whether cellspecific Hi-C data contributes significantly to the model performance as opposed to using averaged Hi-C signals across celltypes. * indicates statistically significant differences using a paired t-test across the six cell types.

13. Page 8, lines 3-11, should be merged with the previous section.

*Reply: Thank you! We have now completely restructured this part of the manuscript and organized this discussion into a results section entitled “Decoding the deep learning models identifies 5hmC features most predictive of gene expression”*

Reviewer #2:

***NOTE:*** *We have colored newly added or modified text with blue font throughout the main text. For this response document, the reviewer comments are in red font, our responses are in black font and quotes and copy/pastes from the manuscript are in blue font.*

1. Why are genes classified as ON/OFF? I think working with signal values in a regression model could be more informative than binarizing the signals.

*Reply: We agree that predicting gene expression values is a more relevant problem, however, doing this with a single track of epigenomic information is unlikely to be competitive with the recent approaches integrating hundreds of such tracks to perform the same task. We hope this work will inspire future work by us and others for the integrated utility of 5hmC with other genomic tracks and DNA sequence to predict gene expression values in a regression setting. Moreover, now we use “High/Low” instead of “ON/OFF” nomenclature.*

2. Are differences between AUC distributions in Fig. 1 significant?

*Reply: Thanks for the suggestion. We have now checked the difference between AUC distribution of our*

*DNN model versus that of each of the other three traditional machine learning models using Wilcoxon signed rank test with continuity correction and have reported that these are all significant differences.*

**Figure 1B.** Area under the Receiver Operating Characteristic Curve (AUC) distribution for our FCDNN model and baseline machine learning models: logistic regression (LRg), random forest (FRo), and SVM. For this analysis, we train one model per each sample while holding out one chromosome for validation/development and one chromosome for testing. Statistical significance testing across different models was performed using Wilcoxon rank sum test with *** indicating a p-value less than 1e-8.

3. Why DNNs are trained up to the epoch 60? I wonder if any early stopping criteria based on validation set have been used to prevent overfitting? How about GCN models?

*Reply: Thank you for your inquiry regarding the training duration of the deep neural networks (DNNs) and the use of early stopping criteria to prevent overfitting. The original GCN publication used up to 1000 epochs. For our task we checked for the combined model trained on merged datasets, we plotted a graph that illustrates the performance of a over 600 epochs (Reviewer Figure 2.1). We observed that the model's loss decreases and accuracy converges to a plateau shortly beyond the 60th epoch. In consideration of this observation and to mitigate the risk of overfitting, the training was deliberately capped at 60 epochs for the combined model. For the models dedicated to individual samples, the training was limited to 40 epochs based on a similar performance assessment. It is our belief that this strategy ensures a robust model while maintaining generalizability by not overextending the training duration.*

**Reviewer Figure 2.1:** Plotting the loss and accuracy across different epochs for training the combined model.

4. What is the 5hmC-enrichment profile per bin in GCN models? Is it a scalar or vector? Fig.

3A shows it as a vector. If so, in what resolution these signals are generated, 100bp?

*Reply: The 5hmC-enrichment profile per bin in our Graph Convolutional Network (GCN) models is represented by two measurements (5hmC IP versus input IP) summarizing the signal across each 10kb node.*

5. Can you also plot Precision-Recall curves in Figs. 3B-C?
6. Are the reductions of AUCs from cell-type-specific to averaged Hi-C seen in Figs. 3B and 3C significant?
7. Again, are the AUPRC and AUROC differences when using 10-nearest bins, as shown in Fig. 3D significant? It would be better to plot the distributions (for example, box plots) instead of a scalar score. The authors should perform rigorous statistical tests in all comparisons of the paper to show the significance scores.

*Reply: Now we have added as part of the main figures the AUPR curves next to the AUC models using both matched and aggregate Hi-C data. This figure is now Figure 4 with relevant subpanels pasted below for reference. As part of this, we also computed the statistical significance of these reductions for both cases (Q6 and Q7).*

*Note that the differences in reported values are due to use of the same seed for pseudo number generation across models to make sure our results are reproducible going forward.*

**Figure 4:** (**B**) Evaluation metrics (ROC and PR curves) for each of the six models trained and tested using a matching set of Hi-C, 5hmC signal and expression information per cell-type. DP and Th2 cell types had the lowest scores, likely due to the low sequencing depth of their Hi-C contact maps. (**C**) AUC and AUPR scores to assess whether Hi-C data contributes significantly to the model performance as opposed to simply using 10 nearest bins to the TSS for each gene (i.e., 5 upstream and 5 downstream bins of TSS). (**D**) Evaluation metrics for each of the six models trained and tested using an averaged set of Hi-C contacts (Hi-C data from each cell type was subsampled to the same number of valid interaction pairs before aggregation) but with cellspecific 5hmC signal. All samples performed better when using cell-type-specific data with the performance gap being higher for cell types with the highest depth Hi-C data (i.e., B cells with 1B+ valid interactions). (**E**) AUC and AUPR scores to assess whether cell-specific Hi-C data contributes significantly to the model performance as opposed to using averaged Hi-C signals across cell-types. * indicates statistically significant differences using a paired t-test across the six cell types.

8. ICE normalization can suffer from distance effect. Have you tried other methods that account for distance effect (like HiC-DC+)? The same issue exists in Fig. 5A. It seems most of the top-10 interactions are the nearby bins. I would suggest creating graphs based on significant interactions of HiC-DC+ and compare the results with the graphs extracted based on top-10 interactions.

*Reply: We thank the reviewer for their suggestion. We indeed repeated our analysis now using most significantly interaction top-10 bins using FitHiC as a tool for significance assignment. This gave us very similar results between GhmCN and FitHiC. The prediction accuracies were nearly identical (Accuracy, AUC, AUPR all within 1% of each other) and when we focused on one of our specific case studies using GNNExplainer on the FitHiC-based Hi-C graph, we got the same 10 regions as prioritized regions for Aicda gene compared to our original GhmCN model with only slight differences in contribution scores.*

**Reviewer Figure 2.2:** Overlap between regions predicted from GhmCN and from ABC-5hmC models for activated B cells.

9. It is not clear how the graphs are built from the Hi-C data. Are these graphs per chromosome? How many nodes and edges are there in each graph? What are the degree distributions of gene and non-gene nodes? This information can help better understanding of the GCN models.

*Reply: We have now added additional description to the Methods section to clarify this. We have also plotted the degree distributions*

*In our methodology, we started by normalizing the Hi-C signal using the ICE algorithm (Imakaev et al., 2012). To further refine this normalized data, we implemented a distance normalization by deducting the median values of the upper diagonals from each data point (negative values are set to zero). Subsequently, we constructed a network model per chromosome wherein each node is connected to its top-10 nearest neighbors, denoted by k = 10. Due to the undirected nature of the network, certain nodes may be connected to more than ten neighbors. This is because a single gene node may rank within the top 10 neighbors for multiple other genes. It is important to note that we experimented with a network of 15 neighbors per node. However, we encountered issues with memory usage, a challenge also highlighted by (Bigness et al., 2022).*

**Reviewer Figure 2.3:** Degree distribution for graph constructed from Activated B cell (72hr) dataset for all nodes (left), nodes with promoters or gene nodes (middle) and for nodes without a promoter (right).

10. The authors refer to GC-MERGE (Bigness 2022) for the GCN models used. It would be helpful to provide a simple schematic of model architecture, inputs, and outputs.

*Reply: We thank the reviewer for this suggestion, we have initially not presented this because of the similarity between our work and GC-MERGE but we now added it in Supp Figure 3A and we agree it was important to include.*

**Supp. Fig 3A:** Schematic representation of the GhmCN model.

11. There is not any comparison between DNN models (which only uses 5hmC signal in the gene bodies and their vicinities) and GCN models (which uses 5hmC signals in all genomic bins connected to the genes based on Hi-C interactions). This is a very interesting benchmark to see how much Hi-C and 5hmC signals in enhancers can add to the predictive power of the models.

*Reply: We thank the reviewer for the suggestion. However, due to difference in scales at which these two approaches operate with (as small as 100bp bins for DNN and 10 kb bins for GhmCN), it is not possible to accurately tease out the contribution of Hi-C data to prediction power by comparative analysis DNN vs GhmCN. Therefore, we assess each approach with different variations and baselines that operate at the resolution matched to that approach. The distinct power of GhmCN, and now the ABC-5hmC approach, is the ability to prioritize putative regulatory elements with respect to their potential effect on their target genes, which is highly valuable as we demonstrate in the manuscript.*

12. Regarding “Since 5hmC is a stable, covalent DNA modification, assessing 5hmC signals would enable the study of such samples and can also provide estimates on differences of gene expression and enhancer utilization across different conditions”, is Hi-C data available in these samples? If not, the proposed models cannot be used to identify the enhancers.

*Reply: You are indeed correct that, although one part of the sentence makes sense, the other part about enhancer utilization is quite an unlikely scenario where one would have Hi-C data from such DNA samples. We now removed the “and enhancer utilization” part from this sentence.*

*However, note that, our manuscript has the analysis that demonstrated the efficacy of using an aggregated Hi-C dataset across cell types. Therefore, it is possible to have a case where 5hmC data is available for a sample and can be used together with Hi-C data from other relevant samples to predict gene expression and to prioritize enhancers. For the sake of brevity, we decided to not discuss this in the specific sentence above as you suggested. In the next sentence, we now discuss the possibility of using 5hmC alone to get some sense of epigenetic gene regulation is such samples as below:*

*Since 5hmC is a stable, covalent DNA modification that survives DNA extraction protocols, assessing 5hmC signals would enable the study of such samples and would also provide estimates of differences in gene expression across different conditions (e.g., stimulated vs unstimulated cells, healthy vs tumor tissue). Given the enrichment of 5hmC in enhancers, and our demonstration that using aggregate contact maps from other relevant cell types is a reasonable approach, 5hmC (CMS-pulldown) measurements alone may be sufficient to provide a glimpse of epigenetic regulation in such samples. Exploration of potential distal regulatory elements and chromatin contacts for such samples would not otherwise be possible. Our study sets the stage for future work that utilizes 5hmC, on its own or in addition to other genomics and epigenomics datasets, for modeling gene regulation.*

Minor comments:

-Page 14, line 32: the yellow highlight cannot be seen in Fig. 5. Was it supposed to be pink?

*Reply: Thanks for noticing this. It was yellow on the UCSC genome browser but is pink or more like purple on the PDF. We have now corrected this to purple.*

-Page 16, line 54: table S8 should probably be table S9.

*Reply: Thanks for catching this. Yes, you are correct and we have now fixed this.*

-Page 14, line 38: H3K27ac is repeated.

*Reply: We have now fixed this.*

Reviewer #3:

***NOTE:*** *We have colored newly added or modified text with blue font throughout the main text. For this response document, the reviewer comments are in red font, our responses are in black font and quotes and copy/pastes from the manuscript are in blue font.*

1. Section "Within sample predictive models of gene expression from 5hmC enrichment" does not give detailed enough information about the model inputs, which are neither clarified in Methods. Specifically, what are the inputs of the different machine learning methods? In Fig. 1A, there are "bins" (?) 1 to 230. What do those represent? Are these genomic bins? If yes, which regions do they refer to?

*Reply: All machine learning methods, namely random forest, logistic regression, SNV, and deep neural networks, utilize the same information encapsulated in the 230 bins. These bins represent genomic regions, as clarified in the Methods section but also in the results discussion as below: For each sample, we obtained 5hmC signal per bin using 5hmC enrichment versus input (normalized for sequencing depth and bin size). For each gene over 1 kb in size (n=21,752), we selected a total of 230 5hmC features using fixed and variable sized bins across the gene body and around the TSS and TTS (****Methods, Supplementary Fig. S1A-B****).*

*Also in results, we now make it explicit that all models use the same input features, expression labels and train/validate/test splits.*

*We then trained FCDNNs for the same predictive task using the same 5hmC input features and the same train/validate/test split. Using the validation set, we first selected hyperparameters such as number of layers and neurons per layer (Table 2). We then compared the resulting FCDNN models and observed that they significantly outperform the three machine learning approaches discussed above (Fig. 1B) with a median AUC of 0.89 across all samples (row “Sample-specific” AUCs in Table 3 and F1 scores in Table 4 with per sample statistics in Table S5 and S6, respectively).*

2. Section "Cross-cell type comparisons with GhmCN" (and also Methods) is lacking information, how exactly information is transferred from one cell type to another. As I understood from the current version of the manuscript, the "input features" also include the graph structure (which includes Hi-C contacts as nodes). If the Hi-C contacts in another cell type are different, how can the network (parameters) be transferred to that cell type?

*Reply: We are sorry for the confusion. This is related to how GraphSAGE operates compared to traditional node representation learning. GraphSAGE, focuses on developing an aggregation function that synthesizes feature information from a node's immediate network vicinity to efficiently produce vector embeddings. These embeddings capture both the structural position and the attributes of the nodes within the graph. Once trained, this function is adept at generating embeddings for novel data, thus extending its utility to unseen datasets, beyond the scope of its initial training.*

3. For case study A, I wondered what we gain from the (rather complex) GhmCN model compared to a simple approach, where we first ranked the Hi-C contacts (as done for the network anyway) and then testing contacting regions of their 5hmC signal. Would such a simple approach be equally suited to propose putative enhancer regions?

*Reply: We thank the reviewer for suggestions of alternative/simpler strategies to achieve what GhmCN is reporting. As part of other reviewers’ comments and as a way to put our work in the context of published approaches, we decided to include a new section and a main figure detailing our adaptation of the Activity-by-Contact (ABC) model to utilize 5hmC signal. Our analysis highlights the utility of ABC-5hmC compared to the classical ABC approach using H3K27ac as Activity signal. Please refer to the section “5hmC-based Activity-by-Contact model identifies novel distal enhancers and their target genes” for the details of this comparison and see below a portion of Figure 3.*

**Figure 3: Activity-by-contact (ABC) model using 5hmC versus H3K27ac.** (**A**) Schematic representation of the published ABC (referred to here as ABC-H3K27ac) and our new ABC-5hmC model. Both models use ATAC-seq peak regions as “candidate enhancers” and same Hi-C data for computing the contact score. (**B**) Venn diagram between ABC-5hmC and ABC-H3K27Ac prioritized regions using data from activated B cells (72hrs). ABC-5hmC captured most of the regions prioritized as putative enhancers by ABC-H3K27Ac.

*Reply: We also assessed the overlap between this rather simple ABC way of utilizing 5hmC signal to prioritize putative regulatory regions versus our GhmCN framework in Supp Fig 3B. Although the two models work at very different scales (ABC-5hmC at ATAC-seq peak level and GhmCN at 10 kb bins), there is a substantial overlap between the two. However, there are over 22k regions that are reported by GhmCN but not by the ABC-5hmC model, one of which is depicted in the Aicda locus example (~160kb region with dynamic gain of 5hmC like validated enhancers). A systematic comparison of these ~22k regions to ABC results remains challenging due to their size differences (e.g., cannot make heatmaps or aggregate signal plots that are comparable).*

**Supp Fig 3B:** Overlap between regions predicted from GhmCN and from ABC-5hmC models for activated B cells.

4. The authors state in the abstract that they "show that 5hmC levels alone can predict gene expression with a predictive power comparable to models that utilize combinations of histone modifications" and in Discussion "Also, our predictive power was comparable or better with respect to existing models that use the genomic distribution of multiple histone marks [...]" However, I did not find a direct comparison to such methods in the current version of the manuscripts. Specifically, the authors report AUC values from previous studies in the Discussion. However, I do not consider these comparable to the AUC values reported in this manuscripts. As the authors show, performance varies greatly even between the cell types studied in this manuscript. In addition, there are additional variables (e.g., discretization thresholds) that might impair a direct comparison. Hence, I consider a direct comparison necessary to make such a statement. Otherwise, the statement (which is in some sense central to this manuscript) is not supported by the data shown.

*Reply: We appreciate the reviewer’s insightful observation regarding the need for a more in-depth and head-to-head comparison to existing methodologies, specifically DeepChrome and AttentiveChrome. The reviewer rightly points out the importance of ensuring comparability in terms of the sets of genes used for training/validation/test. It’s a valid concern whether any observed differences stem from the modeling approach or the data type employed.*

*In response, we would like to clarify that the datasets used by DeepChrome and AttentiveChrome are a number of commonly used human cell lines some of which do not have 5hmC profiles. Fully differentiated cell types and cell lines have very low levels of 5hmC, which makes them not an ideal model for studying the role of 5hmC in gene regulation. Therefore, we chose to remove direct comparisons to DeepChrome and AttentiveChrome in this revision.*

*Nevertheless, we recognize the importance of putting our work in the context more comprehensively. We now included a whole section in the manuscript entitled “Further assessment of our predictive models and potential confounding factors” which also includes a new baseline model that uses a majority voting strategy and gets to memorize (unlike our method) gene expression across training samples. In addition, for the second part, we performed a comparison with the ABC method, and we expanded our work in a new section to provide additional insights into our findings. We believe that this adjustment enhances the clarity and completeness of our comparative analysis.*

5. The discretization approach (as far as I understand) the authors chose (based in the median gene expression) implicitly assumes that genes are "On" in half of the samples/conditions, which will likely be false for cell type-specific genes on the one end of the scale and "house keeping" genes on the other end. If I misunderstood the labeling and this is based on the global median (across all genes and samples), my minor comment regarding RPKM/TPM becomes more relevant.

*Reply: We apologize for this confusion. Indeed, our discretization is neither the first nor the second strategy outlined by the reviewer above. It is simply, for each sample, taking the median across all genes and labeling those below the median as “Low” and above as “High” expression. We now clarified this in the text as below to avoid confusion:*

*To determine the set of genes to be used, we utilized UCSC gene annotations for the Dec. 2011 (mm10) assembly of the mouse genome and excluded genes with size smaller than 1 kb leaving us with 21,752 genes. Data from RNA-seq experiments were then used to define labels for each of these remaining genes using the median TPM value for that sample as a threshold to label genes as either “High” (above median) or “Low” (below median) expression (Fig. S1A).*

**Minor comments:**

6. From my perspective, the first section of Results (Compilation of 5hmC and gene expression datasets) does not present actual results but rather data processing and should be transferred to the Methods section.

*Reply: Thanks for the suggestion. We now moved the bulk of this discussion into Methods but kept brief sentences to refer to Methods in the results section as below*

*After quality control and selection of one representative replicate for each experimental condition, we kept 49 samples to develop our predictive models (****Methods****). …*

*For each gene over 1 kb in size (n=21,752), we selected a total of 230 5hmC features using fixed and variable sized bins across the gene body and around the TSS and TTS (****Methods, Supplementary Fig. S1A-B****). For the same set of genes, we categorized their expression state into two groups (High vs Low) using median value of gene expression for that sample (****Methods,*** ***Supplementary Fig. S1C****).*

7. In some of the sub-figures of Fig. 1, legends are unnecessary, because the same labels are already provided at the x-axis.

*Reply: We thank the reviewer for their suggestion. We try, as much as possible, to have the figures selfexplanatory. This sometimes leads to repetitions like those pointed out. We tried to minimize them in this revision.*

8. In section "Aggregate predictive models of gene expression from 5hmC enrichment" (line 61), the authors state that the use "training and test splits similar to the Whole model." Why did they not use exactly identical splits? If they did, numbers (AUC, F1) would be directly comparable.

*Reply: We thank the reviewer for pointing this out, this is just miswording on our side. The splits were indeed the same, not similar. However, since in this new version, we removed the ESC vs Immuno model distinction, this part of the text also got removed so the issue is not relevant to this revised version.*

9. In Fig. 1C, the authors draw AUC values from ESC cells as red dots over the boxplot for the "Whole" model. For a direct comparison the "ESC" model, an additional boxplot or, even clearer, a scatter plot of "Whole" vs "ESC" AUC values would be more informative.

*Reply: For the reason stated above (removal of ESC vs Immuno model distinction), this part is not relevant anymore. However, we now took the additional step of showing each dot on our boxplots (such as Fig 1B and 1D) to allow for a more informative representation.*

10. In the sub-title of Fig. 1D, the other use the term "cross-validations" (which is typically understood differently in the field), whereas in the text they use "cross-cell type". I would suggest to also use this term in the sub-title of Fig. 1D for clarity.

*Reply: Due to removal of ESC vs Immuno analysis, this subfigure is not there anymore in the manuscript. However, we thank the reviewer for their suggestion on this and we agree it was confusing. We have now modified the text and figures to provide a schematic representation of the training/validation/testing split for all different types of models we have trained. Such schematic representations and rewording of the text, hopefully, clarify most of the confusions.*

11. The authors evaluate their GhmCN models in the cross cell type setting and find that AUC values are substantially worse for some combinations of training and test cell type. I wondered if Hi-C information has been even harmful for model performance in such cases (relative to a model that does not use Hi-C information).

*Reply: This is an interesting question. However, due to difference in scales at which our two approaches operate (as small as 100bp bins for DNN and 10 kb bins for GhmCN), we are not sure if we can satisfactorily answer this question. For instance, it is not possible to accurately tease out the contribution of Hi-C data to prediction power by comparative analysis DNN vs GhmCN due to this scale difference. It is also possible that a good fraction of the Hi-C data (aside from activated and resting cells with 1B+ valid interactions) we are using is not of sufficient depth to identify weaker signals such as enhancer-promoter loops. It is* *reasonable to argue that, for most of the genes, the contribution of proximal 5hmC signal at high resolution could be higher compared to distal interactions (excluding anything less than 10kb apart) identified from Hi-C data. However, the distinct power of GhmCN, and now the ABC-5hmC approach, is the ability to prioritize putative regulatory elements with respect to their potential effect on their target genes, which is highly valuable for studying key cell-type-specific genes and their distal regulation as we demonstrate in the manuscript.*

12. In Fig. S3B/C, we see that the ranking of model performance (AUC) across cell types often coincides with the similarity of their expression profiles. However, this does not seem to be the case for naive CD8T (test) and naive CD4T (train), whereas vice versa the ranking is as expected. Could the authors comment on possible reasons for this observation?

*Reply: This is a good observation and a valid question. We have checked the Hi-C sequencing depth and they were similar between naive CD4 and CD8 T cells. We also checked the 5hmC data quality scores and enrichment, and they were also similar between two cell types.*

*When we look carefully at the actual AUC values rather than ranking across 6 cells, the gap between training on one naive T cell subset and testing on the other was not very large 0.703 vs 0.758. What changed was the improved predictions for CD8 when models were trained on B cells. Interestingly, models trained on naive CD8 T cells also did better for both activated and resting B cell predictions and naive CD4 T cell models consistently perform the worst for B cells. This could be related to some intrinsic properties of CD4 Hi-C data but without any evidence in our hand for this or the contrary, we unfortunately do not have a satisfying answer.*

13. In section "Explaining the predictions of our GhmCN models," the authors state that "the top ranked nodes for each gene showed a high probability of containing regulatory elements with biological significance." However, I did not find any Figure associated with that section that supports this statements Currently, I assume that this is rather related to the structure of the manuscript, and this is shown in the subsequent case studies. Please clarify

*Reply: We thank the reviewer for their attention here. We agree it was mainly about the ordering of sections but now we modified this sentence as below to be more precise and to better connect to the following case studies discussed next:*

*“Through our analysis and prioritization of nodes/regions that interact with gene-containing nodes, we found that a subset of the top ranked nodes for each gene contained regulatory elements with biological significance. This is depicted for two case studies where we focused on wellcharacterized loci harboring key genes for the cell type studied.”*

14. In Fig. 4A (and partly also in Fig. 5) some labels and also the scale (genomic positions) are hard to read and in some cases partly cropped.

*Reply: We have now carefully revised both figures to make sure everything is legible and we used better fonts for the couple of cases where the text looks stretched. Some of these issues may be due to PDF conversion in the Editorial Manager system but we hope the original high-quality figures we upload make it to the reviewer in case this happens.*

15. In case study B, the authors state that "Both ReMap 2022 and UniBind provided further evidence for BATF and IRF4 binding as well as general bZIP TF binding in Kif3a-A and Kif3a-B.", which I could not spot from Fig. 5. Please clarify where that information is represented.

*Reply: We appreciate your attention to detail, and to provide clarity, we acknowledge that the information was edited for visualization purposes. The bold text at the bottom of the figure were indeed obtained from ReMap 2022 and UniBind, supporting the mentioned TF binding.*

*In the revised manuscript, we explicitly mention and clarify that the information from ReMap 2022 and UniBind, specifically related to BATF and IRF4 binding, as well as general bZIP TF binding, was obtained from the bold elements at the bottom of (now) Fig. 6.*

*From the figure caption: These regions also show strong signals of accessibility and some level of active histone marks such as H3K27ac and H3K4me1 in addition to binding of a group of TFs identified using UniBind and ReMap2022 databases (bold text at the bottom).*

16. In Material and Methods, "Analysis of RNA-seq datasets", the authors describe that they used RPKM-normalized expression values. Although it might not make a (major) difference for discretizing based on the median, I wondered why the authors decided against (more established, from my perspective) TPM values.

*Reply: We appreciate your observation and would like to clarify that this was indeed an oversight. In reality, we used TPM (Transcripts Per Million) values, not RPKM. We apologize for any confusion caused by this error, and the text now correctly reflects that TPM values were utilized for the RNAseq part of our analysis. Note that, for summarizing 5hmC signal per bin, we utilized RPKM as stated.*

*Data from RNA-seq experiments were then used to define labels for each of these remaining genes using the median TPM value for that sample as a threshold to label genes as either “High” (above median) or “Low” (below median) expression (Fig. S1A).*

17. In Material and Methods, "Analysis of RNA-seq datasets", they authors term the gene labels as "High" and "Low", whereas in Figures and other parts of the manuscript, these are called "On" and "Off". While I consider the first term more appropriate, I would at least recommend to use consistent labels.

*Reply: Thank you for bringing up this discordance. We have now corrected this to “High/Low” expression throughout the text and the manuscript.*

18. The implementation of Deep Neural Networks (FCDNN) seems to be missing from the github repository.

*Reply: We thank the reviewers for their attention to the missing implementation. We have added to our GhmCN repository a yml file for the GCN, a yml file for the FCDNN, and a folder with yml files for other analysis, such as running the ABC model, performing the DeepLift analysis, performing analysis in R, and more. Moreover, we have deposited into this same repository a folder with the source files to reproduce the FCDNNs.*

19. While the authors explicitly state (Materials and Methods, Deep Neural Networks) that FCDNNs are implemented using TensorFlow (NB: which does not belong, but is only implemented in Python) and Keras, this information (pytorch according to the github repo) is missing for the GCNs.

*Reply: We appreciate your observation and would like to clarify that this is further clarified in the manuscript. In particular, we added the text snippets below:*

*DNN: We developed our DNN models in pyTorch and translated them into Keras for the DeepLift analysis.*

*GCN: The GCN models were implemented using PyTorch and PyTorch Geometric, where the source function for the GraphSAGE operator is found.*

20. Additional details should be given for the application of GNNExplainer to the GCNs. Currently, this is only one sentence in section "Graph Convolution Networks."

*Reply: In response to the request for additional details on the GNNExplainer, we now added a subsection to the Methods as also pasted below.*

***GNNExplainer analysis.*** *GNNExplainer, a framework for interpreting Graph Neural Network predictions, was employed to elucidate the contributions of node interactions within our GhmCN model. We utilized the GNNExplainer function from the torch_geometric.explain library using default parameters and the suggested number of 200 epochs for node-level explanations. We visualize the generated output using the EGA_visualize_subgraph function, which plots the target node together with its prioritized neighbors with edge color (darkness) indicating the order with respect to their significance scores. We explained the queried nodes up to 1-hop away (k-hops = 1).*

21. Typos: Materials and Methods, "Analysis of 5hmC Enrichment datasets," line 28: 1000bpwe -> 1000bp we

*Reply: We appreciate your attention. We revised this typo and thoroughly checked our manuscript for other such cases.*

**Second round of review**

**Reviewer 1**

I would like to commend the authors for their thorough revision of the manuscript and their detailed replies. Our comments have satisfactorily been addressed in this revised version and I only have minor comments left.

1. Last line of the abstract: "feature" should be "future"

2. I wonder about the potential relationship between the expression variability provided in Supp. Fig. 1D and the variability of 5hmC signal. Could the authors provide the standard deviations / confidence interval in Supp Fig. 1A as well?

**Reviewer 2**

The authors have addressed my questions, and I have no further comments.

**Reviewer 3**

The authors have adequately addressed my previous concerns.
I only have one miniscule comment regarding text added to the revised version of the manuscript:

In the new section "Further assessment of our predictive models and potential confounding factors", I was slightly confused by the description of the assessment in different quartiles. Specifically, the authors report accuracy values for Q1 to Q4, where in each quartile genes are predicted as High or Low. If I understood the procedure correctly, all genes in Q1 and Q2 should be predicted as "Low" and all genes in Q3 and Q4 should be predicted as "High". If this is correct, it could be stated explicitly, since the current description let me wonder (initially), why there should be e.g. "Low" genes in Q4.

**Authors Response**

**Point-by-point responses to the reviewers’ comments:**

Reviewer #1:
I would like to commend the authors for their thorough revision of the manuscript and their detailed replies. Our comments have satisfactorily been addressed in this revised version and I only have minor comments left.
- Last line of the abstract: "feature" should be "future".
*Reply: Corrected.*

- I wonder about the potential relationship between the expression variability provided in Supp. Fig. 1D and the variability of 5hmC signal. Could the authors provide the standard deviations / confidence interval in Supp Fig. 1A as well?
*Reply: We have now provided 95% confidence intervals around the mean for this figure in Supp. Fig 1A.*

Reviewer #3:

The authors have adequately addressed my previous concerns. I only have one miniscule comment regarding text added to the revised version of the manuscript: In the new section "Further assessment of our predictive models and potential confounding factors", I was slightly confused by the description of the assessment in different quartiles. Specifically, the authors report accuracy values for Q1 to Q4, where in each quartile genes are predicted as High or Low. If I understood the procedure correctly, all genes in Q1 and Q2 should be predicted as "Low" and all genes in Q3 and Q4 should be predicted as "High". If this is correct, it could be stated explicitly, since the current description let me wonder (initially), why there should be e.g. "Low" genes in Q4.
*Reply: We apologize for the confusion but your understanding is correct. We have now added “Q1 and Q2 are genes labeled with “Low” expression state, whereas Q3 and Q4 are genes labeled with “High” expression state.” to the figure caption.*
